# Rising food prices and widening nutrition gaps in Mexico: The cost of nutrient-dense and ultra-processed foods before, during, and after COVID-19

**DOI:** 10.1371/journal.pone.0355198

**Published:** 2026-08-26

**Authors:** Alfonso Mendoza-Velázquez, Jonathan Lara-Arevalo, Leonardo Mendoza-Martínez, Adam Drewnowski

**Affiliations:** 1 Centro de Investigación e Inteligencia Económica, Universidad Popular Autónoma del Estado de Puebla (UPAEP), Puebla, México; 2 Center for Public Health Nutrition, University of Washington, Seattle, Washington, United States of America; 3 Department of Nutrition, Gillings School of Global Public Health, University of North Carolina at Chapel Hill, Chapel Hill, North Carolina, United States of America; 4 Department of Economics, Universidad de las Américas Puebla (UDLAP), Puebla, México; Lusofona University of Humanities and Technologies: Universidade Lusofona de Humanidades e Tecnologias, PORTUGAL

## Abstract

The COVID-19 pandemic disrupted global food systems, contributing to rising food prices and altering the affordability of healthy diets. This study examined the relationship between nutrient density of foods, food prices and inflation in Mexico before, during, and after the pandemic. Foods from the ENSANUT dietary survey were assigned to 4 categories using the NOVA classification system and were linked to nutrient composition data and national food price series from 2012 to 2025. The Nutrient Rich Food Index (NRF9.3) was a measure of nutrient density. Affordability was defined as nutrient density relative to price per 100 kcal. Trends in prices and inflation were analyzed using a panel data model with food-level fixed effects. Minimally processed foods had substantially higher nutrient density (NRF9.3: 202.2) than processed (15.7) and ultra-processed foods (UPFs; 5.4), but were also markedly more expensive per 100 kcal. Across the study period, higher nutrient density was consistently associated with higher prices per calorie. During the pandemic, inflation for minimally processed foods increased by 1.64 percentage points (p.p.; p = 0.064) relative to pre-pandemic levels, and by 6.04 p.p. in the post-pandemic period (p < 0.001). Processed foods and culinary ingredients experienced additional inflation of 1.99 p.p. and 1.21 p.p., respectively, during the pandemic, while no significant differences across processing categories were observed post-pandemic. Following the onset of the pandemic, food prices increased across all processing categories. Because minimally processed foods had higher baseline prices, similar inflation rates translated into a widening affordability gap between more and less nutritious foods. These findings suggest that foods aligned with dietary recommendations became relatively less affordable during a period of economic disruption, potentially incentivizing substitution toward lower-cost, energy-dense UPFs. Policies that improve the affordability and price stability of nutrient-dense foods may be critical to support healthy diets under conditions of economic volatility.

## Introduction

The COVID-19 pandemic disrupted food value chains around the world. During the initial phases of the pandemic, containment measures adopted by national governments to reduce infection rates caused disruptions that affected all stages of the food supply—from production and processing to distribution and retail—leading to rising food prices and inflation [1]. Reports from the United Nations’ Food and Agriculture Organization (FAO) documented substantial increases in global food prices both prior to and especially during the two-years of the pandemic [2]. According to the International Monetary Fund (IMF), the Global Food Price Index increased by 77.26% in April 2022 compared to March 2020, at the onset of the pandemic [3]. Although some market correction followed, food prices continued to rise [4–6], further exacerbated by armed conflicts, droughts, and extreme weather events. These economic trends have disproportionately affected populations of low- and middle-income countries (LMICs) [7,8].

Food prices in Mexico, before, during and after the pandemic provide a relevant case study. Food consumption patterns in Mexico, as in other LMICs, are strongly driven by food prices and by consumer socio-economic status [9]. In general, fresh meat, dairy, vegetables and fruit cost more per 100 kcal compared to grains, fats and sweets [9–12]. Lower-cost, energy-dense foods providing more calories per peso were more likely to be consumed by lower-income and rural populations [9]. In addition, COVID-related price increases have not been uniform across food groups. Prices of fresh and perishable foods (e.g., meat, poultry, fish, and fruits) have risen more sharply than those of discretionary foods [10]. This has led to concerns that nutritious diets based on minimally processed foods may become increasingly difficult to afford, not only for the global poor but also for middle-income populations [13].

The well-known NOVA classification system [14,15] assigns foods into 4 categories based on the degree of industrial processing: minimally processed foods, processed foods, ultra-processed foods (UPFs), and culinary ingredients [14]. In general, UPFs receive lower nutrient density scores compared to minimally processed foods [11]. A higher consumption of products from the UPF category, which includes many packaged foods high in refined grains, added sugars, sodium, and vegetable fats [14,16], has been associated with adverse health outcomes [14,16].

The FAO has emphasized the importance of affordable nutrient density, underscoring the need to identify foods that are both nutrient-rich and economically accessible [17]. UPFs are typically less expensive sources of dietary energy, often costing less per 100 kcal than minimally processed foods [11,18–21]. However, the interaction between the NOVA level of processing, nutrient density, food prices and food inflation has not been sufficiently examined. While the low cost of UPFs has been documented, it has been less frequently integrated with analyses of nutrient density and price dynamics over time.

The present goal was to examine nutrient density and changes in food prices and inflation by NOVA category in Mexico before, during, and after the COVID-19 pandemic. Given that food processing can enhance shelf stability and supply chain resilience [22,23], we hypothesized that minimally processed foods would exhibit greater seasonal variability and sharper price increases compared to processed foods, UPFs, and culinary ingredients. The findings are relevant for dietary guideline development and for policymakers addressing food insecurity and diet-related chronic disease in LMIC settings.

## Materials and methods

### The ENSANUT 2018 food composition database

The National Health and Nutrition Survey (ENSANUT) is a nationally representative survey of diets and health in Mexico including modules on food insecurity, dietary intake and health outcomes [24]. Dietary intake data are collected using both a Food Frequency Questionnaire (FFQ) and a 24-hour recall. The present study used the nutrient composition database for 157 food products included in the ENSANUT FFQ. Each food item contained information on at least 12 nutrients of interest. Foods were assigned into 11 food groups. Herbs and spices were assigned into a non-food category along with non-reconstituted coffee powder, low calorie sweetened candy, and chewing gum. Foods with energy density <10 kcal/100 g were excluded for cost and nutrient density analyses because the NRF9.3 is a nutrient-to-calorie ratio, and foods with zero or very low energy values (e.g., water, diet beverages, unsweetened coffee and tea) can lead to division by zero or produce disproportionately high nutrient density estimates due to the small denominator. The ENSANUT food database was merged with data on average food prices and food price indices provided by the National Institute of Geography and Statistics (INEGI).

### NOVA classification

**Fig 1:**
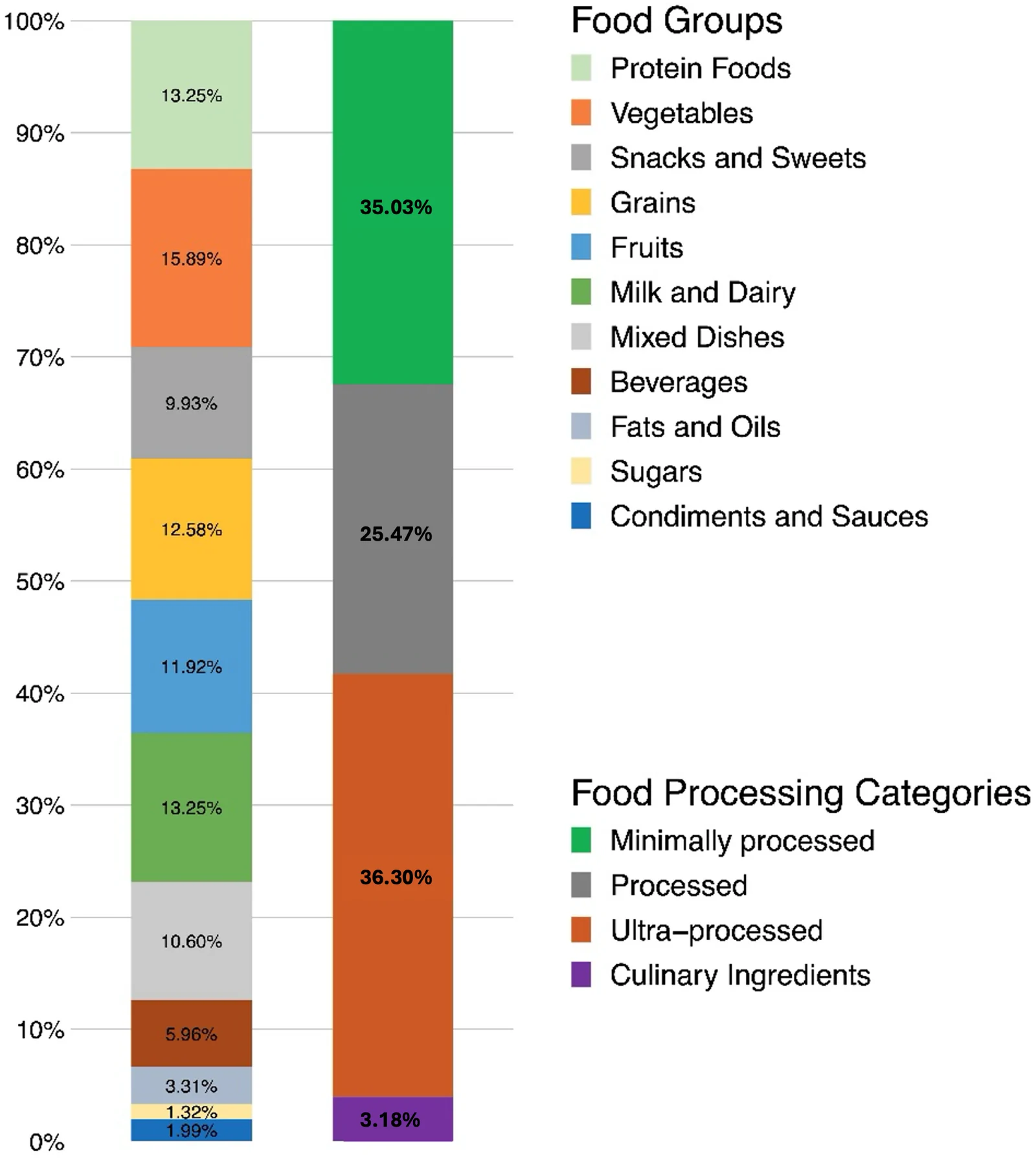
Percent distribution of ENSANUT foods by food groups and NOVA food processing categories.

The NOVA system assigns foods into four categories: minimally processed, processed, ultra-processed and culinary ingredients, based on the purpose and intent of industrial processing [16]. Foods such as fruits, vegetables, grains or meats, fresh milk, and plain yogurt, freshly squeezed juices, eggs, legumes, fish and other seafood, and unsalted nuts and seeds listed by ENSANUT were classified in the minimally processed category. Culinary ingredients included salt, sugar and molasses, honey, syrup, vegetable oils, animal fats (lard, butter), additives, vinegar with additives and preservatives and starches. Processed foods included products combining culinary ingredients with minimally processed foods. Finally, ultra-processed foods (UPFs) are industrial formulations often containing high levels of added sugars, sodium, and fats, as well as additives not used in normal kitchens, such as antioxidants, flavors and stabilizers [15]. Items classified into this category included commercial breads, ready-to-eat breakfast cereals, cakes, sweet snacks, pizzas, French fries, ice cream and frozen meals. Fig 1 shows the distribution of ENSANUT foods by food group (A) and each NOVA category (B).

### Food prices and food price trends

Following past procedures, average food prices for 157 items listed in ENSANUT’s 2018 FFQ were obtained from the National Institute of Geography and Statistics (INEGI) for the month of May 2012 and were expressed in Mexican Pesos (MXN) per 100 g or 100 ml, and per 100 kcal. Average prices were then yield-adjusted to account for preparation and waste. Monthly food price series from June 2012 through July 2025 were constructed using INEGI food price indices. All prices were inflation-adjusted using official food price indices.

Monthly price indices were available for 81 foods or food groups. These indices were applied to the 157 foods in the ENSANUT FFQ such that the same index was applied to foods within a given food group. For example, the same milk price index was applied to various types of milks represented in ENSANUT: whole milk, skimmed milk, semi-skimmed milk, and social assistance milk. Other food groups sharing the same price index were varieties of yogurt (i.e., natural, with fruit, skimmed, drink, danonino, etc.); cheese (i.e., fresh and Manchego); bananas; canned fruits; chicken and parts of chicken; beans; breads; cereals; corn foods; meat; and some fruit groups. Furthermore, some food price indices were available only from August 2018 by INEGI, coinciding with the new consumer price index methodology [25]. To complete the time series of prices for these foods from May 2012, we employed the monthly discount price factors of foods that showed the highest correlation with the respective food from August 2018, computing each monthly price backwards to May 2012. This procedure allowed reconstruction of complete monthly price series for all foods over the full study period. To control for the effect of food-price determinants we also obtained data on gas prices, exchange rates, international cereal and fuel prices from the Federal Reserve Economic Data (FRED) database [26].

### Nutrient density evaluation

The Nutrient Rich Food (NRF) model was used to assess nutrient density of foods. The NRF9.3 score is computed as the difference between a positive nutrient subscore (NR9) and a limiting nutrient subscore (LIM3). The positive NR9 subscore is based on the percentage of daily values (%DV) per 100 kcal for nine nutrients: protein, fiber, vitamin C, potassium, vitamin A, calcium, iron, magnesium, and vitamin D. The negative LIM subscore is based on the percentage of maximum recommended values (MRV) per 100 kcal for three nutrients to limit: saturated fat, added sugar, and sodium [27]. In both cases, nutrient contributions are capped at 100% of the reference value. The NRF9.3 was calculated as:


$$\begin{array}{c} NR\text{9}=\left(\frac{Nut_{inc_{\text{1}}}}{DV_{\text{1}}}+\frac{Nut_{inc_{\text{2}}}}{DV_{\text{2}}}+\ldots +\frac{Nut_{inc_{\text{9}}}}{DV_{\text{9}}}\right) \end{array}$$

$$\begin{array}{c} LIM\text{3}=\left(\frac{{Nut\_\text{lim}}_{\text{1}}\;}{{MRV}_{\text{1}}}+\ldots +\frac{{Nut\_\text{lim}}_{\text{3}}}{{MRV}_{\text{3}}}\right) \end{array}$$

$$\begin{array}{c} NRF\text{9}.\text{3}=\left[\sum_{i=\text{1}}^{\text{9}}\frac{Nut\_inc_{i}}{\left({DV}_{i}\right)}-\sum_{j=\text{1}}^{\text{3}}\frac{{Nut_{\text{lim}}}_{j}\;}{\left(MRV_{j}\right)}\right]x(\text{1}\text{0}\text{0}/energ\_dens) \end{array}$$

Nutrient standards were based on recommendations from the Mexican National Institute of Public Health (INSP) and the Codex Alimentarius [28]. The reference daily values (*DVi*) were protein (50 g), fiber (25 g), vitamin A (800 RAE), vitamin C (100 mg), vitamin D (10 mcg), calcium (1000 mg), iron (18 mg), potassium (3500 mg) and magnesium (310 mg). For nutrients to limit, maximum recommended values (*MRVj*) were: saturated fat (20 g), added sugar (50 g) and sodium (2000 mg) [28].

Affordable nutrient density, defined provisionally as NRF9.3 points by energy cost was calculated per food and per NOVA food group. Time trends for affordable nutrient density were calculated per month over the time period employing the following formula:


$$\begin{array}{c} {Afford}_{it}=\frac{NRF\text{9}.\text{3}}{\left(\frac{MXN\$}{\text{1}\text{0}\text{0}\;kcal}\right)} \end{array}$$

The NRF model has been widely applied in food and diet quality research [29], and has been used in LMIC contexts to assess the nutritional value of foods relative to cost [30,31]. Nutrient profiling tools such as the NRF are particularly relevant in LMIC settings where overweight and obesity coexist with micronutrient deficiencies, including inadequate intakes of vitamin A, B vitamins, folate, calcium, iron, iodine, and zinc [32].

### Statistical analysis

Monthly prices and annual inflation rates for 157 foods listed in ENSANUT 2018 FFQ were calculated for each food for the time series period from May 2012 through July 2025. We leverage temporal variation spanning the COVID-19 pandemic to examine changes in food price dynamics over time. To investigate the effects associated with the COVID-19 pandemic on food inflation per 100 kcal, we estimated a panel data model with food-level fixed effects, month fixed effects, and time-varying macroeconomic controls. Two period indicators (shift dummies) were included: one for the COVID-19 period (March 2020 to February 2022) and another for the post-COVID-19 period (March 2022 to July 2025), with the pre-COVID-19 period as the reference category. To account for seasonality, we included month fixed effects (seasonal dummies). In addition, we controlled for time-varying macroeconomic factors that may influence food price inflation, including gas prices, exchange rates, international cereal prices, and fuel prices.

We conducted a Hausman test, which rejected the random-effects specification in favor of a fixed-effects model. The fixed-effects specification accounts for time-invariant unobserved heterogeneity across foods by allowing each food item to have its own intercept, thereby reducing omitted variable bias and eliminating potential correlation between individual effects and regressors.

The specific fixed-effects model is as follows:


$$\begin{array}{c} ln\left(\frac{p_{it}}{p_{it-\text{1}\text{2}}}\right)=\beta_{\text{0}}+\beta_{\text{1}}Covid_{t}+\beta_{\text{2}}PostC_{t}+\sum_{k=\text{2}}^{\text{4}}\delta_{k}Covid_{t}\times Nova_{k}+ \end{array}$$

$$\begin{array}{c} +\sum_{k=\text{2}}^{\text{4}}\omega_{k}{Post}_{t}\times Nova_{k}+\beta_{\text{3}}t+\sum_{m=\text{1}}^{\text{1}\text{1}}\lambda_{m}D_{m,t}+X_{t}^{\prime }\theta +\alpha_{i}+\epsilon_{it} \end{array}$$

where $\beta_{\text{0}}$ represents baseline inflation for minimally processed foods;$\;\beta_{\text{1}}$ and $\beta_{\text{2}}\;$capture differential changes in inflation for minimally processed foods during and after the COVID-19 period, respectively; $\delta_{k}$ and $\omega_{k}$ capture differential changes in inflation across NOVA categories during and after COVID 19, relative to minimally processed foods; $\beta_{\text{3}}$ captures linear time trends; $\lambda_{m}$ measures monthly seasonal effects; θ is a vector of parameters associated with time-varying macroeconomic controls (gas prices, exchange rates, cereals and fuel prices); $\alpha_{i}$ are food-specific fixed effects; and $\epsilon_{it}$ is the disturbance term. The base period is pre-COVID-19. The interaction terms between period indicators and NOVA categories allow estimation of differential inflation responses by degree of processing, relative to minimally processed foods. Standard errors were clustered at the food level to account for serial correlation within food items over time, and statistical significance was assessed using t-tests, p-values, and Wald tests. The analytic code used for the analyses is available in S1 File.

## Results

Foods in the ENSANUT database (n = 157) were categorized into food groups following the What We Eat in America (WWEIA) coding scheme [33]. Fig 1 shows the distribution across food groups: vegetables (15.9%); milks and dairy (13.3%); proteins (13.3%); grains (12.6%); fruits (11.9%); mixed dishes (10.6%); snacks and sweets (9.9%); beverages (5.9%); fats and oils (3.3%); and sugars (1.3%). According to the NOVA classification, 55 foods (35.0%) were minimally processed, 40 (25.5%) were processed, 57 (36.3%) were ultra-processed foods (UPFs), and 5 (3.2%) were culinary ingredients.

### Energy and nutrient density by NOVA category

Table 1 shows energy density (kcal/100 g), values of the NRF9.3 nutrient density score, prices per 100 kcal, and our affordability measure for NOVA categories. Minimally processed foods had the lowest energy density (77.29 kcal/100 g), followed by processed foods (174.53 kcal/100 g) and UPFs (305.57 kcal/100 g). Culinary ingredients had an energy density of 292.92 kcal/100 g. Nutrient density was substantially highest in minimally processed foods (NRF9.3 = 202.2), compared with processed foods (15.7) and UPFs (5.4), while culinary ingredients had intermediate values (31.4).

**Table 1:** Energy density, nutrient density (NRF9.3), food prices (MXN$ per 100 kcal) and affordability metrics by NOVA categories.

				MXN$/100 kcal			Affordability^b^		
Food Category	Energy Density	Nutrient density NRF9.3	LIM^a^	Pre-Pandemic	Pandemic	Post-Pandemic	Pre-Pandemic	Pandemic	Post-Pandemic
Minimally processed	77.29 (12.56)	202.20 (134.42)	0.05 (0.01)	28.89*** (0.38)	37.36 *** (0.31)	45.18*** (0.45)	13.34*** (3.90)	11.34* (3.29)	8.62** (2.57)
Processed	174.53 (15.49)	15.65 (5.24)	0.14 (0.02)	3.19*** (0.04)	4.30*** (0.04)	5.34*** (0.05)	9.38*** (3.49)	7.79** (2.81)	6.23* (2.30)
Ultra-Processed	305.57 (31.52)	5.41 (3.52)	0.14 (0.01)	4.86*** (0.06)	6.43*** (0.05)	8.10*** (0.09)	5.08** (2.79)	4.61** (2.50)	3.74 (2.08)
Culinary Ingredients	292.92 (72.53)	31.42 (35.22)	0.12 (0.04)	1.11*** (0.01)	1.47*** (0.004)	1.86*** (0.03)	3.81 (9.56)	3.15 (8.43)	1.87 (6.29)

^a^LIM refers to the three nutrients to limit: saturated fat, added sugar, and sodium. ^b^Calorie adjusted nutrient points per Peso: Affordability = NRF9.3/ MXN$/100 kcal. Table presents mean values and standard errors. *, **, *** significant at the 10%, 5% and 1% levels, respectively.

### Cost per 100 kcal and inflation rates by NOVA category and nutrient-dense foods

**Fig 2:**
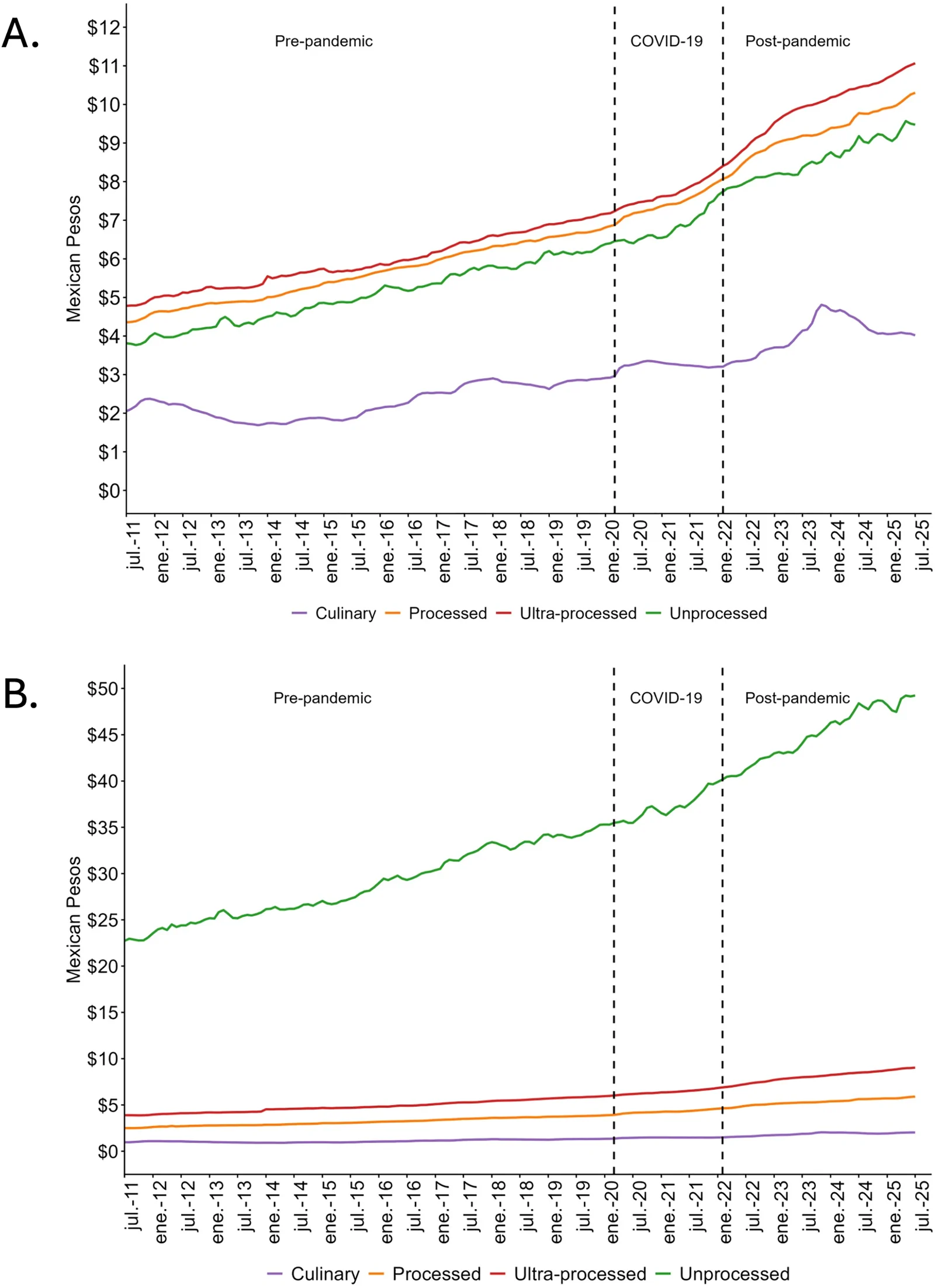
Monthly average prices by NOVA categories. (a) Prices per 100 grams; (b) Prices per 100 Kcal.

Fig 2 shows monthly average prices by NOVA categories for the full sample. Table 1 indicates a persistent and stable price hierarchy across all time periods, with minimally processed foods being the most expensive per 100 kcal, followed by UPFs, processed foods, and culinary ingredients. This ranking remained unchanged before, during, and after the COVID-19 pandemic, despite general increases in food prices across all categories. Across the study period, prices per 100 kcal increased in all NOVA groups following the onset of the pandemic. Minimally processed foods rose from MXN$28.89 in the pre-pandemic period to MXN$37.36 during the pandemic and MXN$45.18 in the post-pandemic period. Although processed foods, UPFs, and culinary ingredients also experienced steady price increases over time, they remained substantially cheaper per unit of energy throughout the entire period. A similar pattern was observed for nutrient affordability (NRF9.3 per MXN$). Minimally processed foods consistently provided the highest nutrient density per peso, although this advantage declined over time (13.34 pre-pandemic; 11.34 during COVID-19; 8.62 post-pandemic). Processed foods and UPFs showed lower and declining absolute nutrient affordability ratios, indicating a widening gap in nutrient return per unit of expenditure across levels of processing.

S1 Table lists the 30 foods in the highest quantile of NRF9.3 nutrient density, which included minimally processed, processed foods, and a few UPFs. Minimally processed foods included spinach, nopal, squash, broccoli, lettuce, several types of chilies, green and red tomato, limes, papaya, green bean, fruit water, strawberry, and zucchini. Foods with high NRF9.3 scores were also identified in processed foods (e.g., canned green chili) and a few UPFs (e.g., cereals, soy, and chocolate powder beverages).

During the COVID-19 pandemic, minimally processed nutrient-dense foods experienced significant price inflation. Limes had the highest rates of monthly annualized inflation (51.80%), followed by squash (23.22%), chicken parts (15.52%), strawberry, melon and fruit water (12.61%), green bean (11.27%), guava (9.70%), cabbage and lettuce (9.36%), nopal (11.84%), and grapefruit (9.27%). Foods with lower inflation during the pandemic were red tomato (−32.86%), green tomato (−29.01%), zucchini (−4.67%), fresh chili (0.09%), poblano chili (2.60%), and pineapple (3.73%). The foods with the highest swings across time were squash, green tomato, chicken-parts, limes and red tomatoes. The steepest falls during the pandemic were shown by red tomato (–32.86%) and green tomato (–29.01%).

As reported in Table 2, UPFs showed, on average, lower rates of inflation than minimally processed foods for the whole period, the pre-pandemic, during and post-pandemic periods. This ordering pattern of minimally processed foods costing more than UPFs is sustained during the three subperiods considered.

**Table 2:** Inflation rates and changes during the COVID-19 pandemic in NOVA price inflation per 100 kcal.

			COVID-19					
	Whole period		Pre-Pandemic		Pandemic		Post-Pandemic	
NOVA Category (nT)	Mean	(SE)^a^	Mean	(SE) ^a^	Mean	(SE) ^a^	Mean	(SE) ^a^
All foods	6.98*	0.08	5.74*	0.10	7.73*	0.19	9.39*	(0.15)
Minimally processed	8.38*	0.19	7.37*	0.25	8.13*	0.49	10.86*	(0.40)
Processed	6.52*	0.11	5.08*	0.14	8.67*	0.29	8.63*	(0.20)
Ultra-Processed	6.11*	0.04	4.85*	0.04	6.68*	0.11	8.67*	(0.10)
Culinary Ingredients	6.42*	0.50	4.96*	0.62	8.06*	0.95	8.86*	(1.14)

* significant at the 1% level. a. Standard error. Inflation estimates represent annualized percentage changes in food prices derived from monthly price indices.

### Changes in food inflation during the COVID-19 pandemic

Table 3 presents panel-data estimates with food and month fixed effects, controlling for unobservable persistent heterogeneity and for common macroeconomic shocks including gas prices, exchange rates, international cereal and fuel prices. The model assessed annual inflation rates of foods in Mexico before, during, and after the COVID-19 pandemic. The model shows that during the pandemic, annual inflation rates of minimally processed foods were higher by 1.64 percentage points (p.p.) relative to the pre-pandemic period, although this estimate is only marginally statistically significant (p = 0.064), and were higher by 6.04 p.p. in the post-COVID-19 period (see β_1_ and β_2_ estimates). With respect to the degree of processing, during the pandemic period, processed foods and culinary ingredients experienced significantly higher inflation relative to minimally processed foods, while no statistically significant difference was observed for UPFs (see *δ* interaction estimates in the table). Processed foods, UPFs, and culinary ingredients had an additional 1.99 p.p., 0.48 p.p. (non-significant), and 1.21 p.p. increase relative to minimally processed foods, respectively. In contrast, for the post-pandemic period, processed foods, UPFs, and culinary ingredients did not show significant differences in inflation relative to minimally processed foods. We observed no differential changes per degree of NOVA processing during this post-pandemic period, suggesting that inflationary changes were similar across processing categories.

**Table 3:** Changes in food inflation during the COVID-19 pandemic (fixed effects GLS estimations, May 2012 to July 2025).

Variable	Parameter	Estimate	Robust Std. errors*	t	P > |t|	[95% IC]
*COVID-19 pandemic*						
Pre	$\beta_{\text{0}}$	18.09	(38.85)	0.47	0.642	(−58.64, 94.829)
During	$\beta_{\text{1}}$	1.64*	(0.88)	1.86	0.064	(−0.10, 3.37)
Post	$\beta_{\text{2}}$	6.04***	(1.69)	3.58	0.000	(2.70, 9.37)
*Interactions during COVID-19*						
Processed	$\delta_{\text{2}}$	1.99***	(0.76)	2.63	0.009	(0.50, 3.49)
Ultraprocessed	$\delta_{\text{3}}$	0.49	(0.65)	0.75	0.457	(−0.80, 1.77)
Culinary	$\delta_{\text{4}}$	1.21**	(0.56)	2.16	0.033	(0.10, 2.33)
*Interactions Post-COVID-19*						
Processed	$\omega_{\text{2}}$	0.53	(0.75)	0.70	0.483	(−0.95, 2.00)
Ultraprocessed	$\omega_{\text{3}}$	0.94	(0.62)	1.52	0.131	(−0.28, 2.16)
Culinary	$\omega_{\text{4}}$	0.78	(0.92)	0.84	0.400	(− 1.04, 2.60)
Trend	$\beta_{\text{3}}$	−0.05***	(0.02)	−2.63	0.009	(−0.08, −0.01)
*Control variables (in ln)*						
Gas price	$\theta_{\text{1}}$	−18.68***	(5.77)	−3.24	0.001	(−25.96, − 3.24)
Exchange rate	$\theta_{\text{2}}$	22.03***	(4.85)	4.54	0.000	(12.45, 31.65)
Cereals price	$\theta_{\text{3}}$	3.07**	(1.19)	2.58	0.011	(1.13, 5.75)
Fuel price	$\theta_{\text{4}}$	0.70	(0.87)	0.80	0.422	(− 1.02, 2.41)

Notes: R2(overall)=0.0206; N = 24,806; n = 157; t = 158. * Clustered standard errors by food. Pre-COVID: May 2012-February 2020; COVID-19: March 2020-February 2022; post-COVID-19: March 2022-July 2025. Variation between ($\sigma_{u}$)=1.31; variation within ($\sigma_{e}$)=11.77; rho = 0.012; Hausman test p-value<0.01. *, **, *** significant at the 10%, 5% and 1% levels, respectively. Inflation estimates represent annualized percentage changes in food prices derived from monthly price indices.

The control variables show that while the price of cereals had a positive and statistically significant effect on inflation, fuel prices did not show a significant association. Exchange rates exert a much greater and significant positive association, with an estimated elasticity of 22.03%, while gas prices show a large and significant negative association (−18.68%), which may reflect the controlled price policy implemented by the Mexican government. The time trend shows an additional small but significant decreasing trend in inflation (0.05 pp per period).

## Discussion

This study examined the relative prices and price inflation for healthy (i.e., predominantly minimally processed) and less healthy (i.e., predominantly UPFs) foods in Mexico before, during, and after the COVID-19 pandemic, providing insight into how food price dynamics evolved across nutrient density levels and NOVA categories over this period.

Three principal findings emerged. First, minimally processed foods had substantially higher nutrient density scores than processed foods and UPFs. Second, foods with higher nutrient density scores also cost more per 100 kcal. Third, foods in the UPF category had lower nutrient density scores but also cost less per 100 kcal.

While minimally processed foods exhibited higher inflation rates in the pre-pandemic period, inflation during and after the COVID-19 pandemic affected all NOVA categories similarly. However, since minimally processed foods cost more per 100 kcal at baseline, these parallel inflationary increases translated into a persistent affordability gap. These findings suggest that the recommended healthy foods continue to be less affordable during a period of macroeconomic instability. The nutrition gap is driven by both higher baseline price levels for healthier foods and by sustained inflationary pressures across all food categories.

Consequently, households with limited food budgets may be driven to purchase lower-cost, energy-dense foods with lower nutritional value [11,34,35]. Mexico is already facing a double burden of malnutrition, characterized by a rising prevalence of overweight and obesity alongside persistent micronutrient deficiencies [36–39]. The observed price dynamics may help explain the current dietary inequalities and worsening nutrition-related health outcomes [40].

### Minimally processed foods are more nutrient dense but more costly on an energy basis

This study confirms past observations [11,19] that minimally processed foods are more nutrient dense than processed foods and UPFs. Minimally processed meat, poultry, fish and fresh produce are rich in protein, fiber and essential micronutrients [41,42]. By contrast, most UPFs are energy-dense and higher in added sugars, sodium, and unhealthy fats [11,16]. Previous research using nutrient profiling models such as the NRF has similarly demonstrated that nutrient density declines as the degree of processing increases from minimally processed to processed and UPFs [11,19].

Relatively few studies have examined UPF nutrient density and cost. Indeed, dietary guidelines emphasize the health benefits of minimally processed foods, while economic constraints may influence the feasibility of these recommendations for households. By integrating nutrient density with price and inflation data, the present study provides a more policy-relevant assessment of food environments, highlighting the structural barriers to accessing healthier diets. Our findings are consistent with prior analyses of US-based data on nutrient density and food prices, showing that nutrient-dense foods cost more per 100 kcal [43]. Studies examining the cost of daily diets similarly report that nutrient-rich dietary patterns more aligned with national dietary guidelines are more expensive, whereas diets higher in added sugars and fats tend to be lower in cost [44,45].

The cost of diets can be captured per 100 kcal or per food unit. Cost per 100 kcal captures the economic trade-offs faced by food-insecure households seeking to meet energy needs at the lowest possible cost [46]. Foods that deliver the lowest-cost calories are typically refined grains, fats, and UPFs. In contrast, cost per gram or per serving may lead to different conclusions [46,47], as many minimally processed foods with high water content (e.g., fruits and vegetables) appear less expensive despite providing fewer calories. This distinction helps explain why healthy foods are not necessarily costlier per unit weight or per serving but are more costly per 100 kcal. From a public health perspective, cost per calorie and cost per nutrient are more informative metrics than cost per gram, as they better capture the ability of diets to meet both energy and nutritional requirements [46].

To complement this perspective, we also examined nutrient density per unit cost (using our affordability metric), an approach aligned with prior work [41,48,49]. This metric adjusts nutrient density by energy cost, providing an estimate of nutrients obtained per peso while holding energy constant. Minimally processed foods scored higher on this metric than processed foods and UPFs, indicating that they provide more nutrients per peso. However, that does not mean that minimally processed foods are inexpensive. Under budget constraints, households often prioritize low-cost calories over micronutrient adequacy [18]. Because many nutrient-dense foods cost more per 100 kcal, they may remain less accessible to lower-income populations despite offering greater nutritional value.

The economic distinctions are critical when it comes to public health guidance. The affordability metric captures nutrient efficiency conditional on energy, but households make decisions based on total food budgets. As a result, even if minimally processed foods provide more nutrients per peso, their higher absolute cost per calorie may still limit their consumption, particularly among lower-income populations. This highlights a central tension in food systems: foods that are affordable sources of energy are not necessarily affordable sources of high-quality protein and key micronutrients. Low-cost diets can alleviate hunger but may fail to meet micronutrient requirements, contributing to hidden hunger and poor diet quality [50].

Previous studies have looked at the relation between nutrient density and cost. For example, dairy products are among the lowest-cost sources of calcium [42], legumes provide inexpensive sources of fiber [51], and fruits are relatively efficient sources of vitamin C [48]. At the same time, UPFs remain among the lowest-cost sources of dietary energy [11]. An important implication is that improving diet quality may require shifting both relative prices and absolute affordability, not only identifying nutrient-dense foods but ensuring that they are financially accessible.

### Inflation affected food prices across NOVA categories

Food prices increased across all NOVA categories following the onset of the COVID-19 pandemic, consistent with global data [52]. However, our panel models indicate that the largest differences in inflation across NOVA categories were observed in the pre-pandemic period. During the pandemic and post-pandemic periods, inflationary pressures were broadly shared across the four NOVA categories.

That said, minimally processed foods continued to exhibit greater price variability and volatility, including sharper spikes and seasonal fluctuations. This greater variability may reflect their dependence on perishable supply chains and exposure to shocks in production and distribution systems [53]. In contrast, processed foods and UPFs, which benefit from longer shelf life and more complex supply chains, showed more stable price dynamics over time. Because minimally processed foods started from a much higher price per 100 kcal, similar inflation rates across categories translated into absolute higher costs, reinforcing existing affordability gaps.

Importantly, food price increases were in excess of inflation rates from the INEGI’s National Consumer Price Index (INPC) [25]. For example, on average, the price increase for minimally processed foods was 8.4% and for UPFs was 6.1%, compared to an average monthly INPC-inflation rate of 5.0%. These findings, along with a deterioration in purchasing power during the pandemic period, may help explain the rising levels of food insecurity reported in Mexico [54].

### Nutrition economics and public health in Mexico

The cost of a healthy diet is reported to be higher in Latin America and the Caribbean compared to any other region in the world [2]. The Consumer Price Index of Latin American countries showed the highest increase (23.5%) between December 2020 and December 2021, compared to 14.5% in Africa and 14.8% in Asia [2]. Foods rich in nutrients of relevance to Mexico and other LMIC, such as iron, vitamin A, calcium and potassium [55] tend to be more costly. Over the past several years, a drop in the consumption of minimally processed foods [56] has been paralleled by a rise in the consumption of UPFs. During the same period, obesity prevalence has risen substantially [57]. The economic disruptions associated with the COVID-19 pandemic may have exacerbated these trends.

Even though the price of nutrient-rich foods did not show a differential increase during the pandemic, those foods were more expensive per 100 kcal to begin with. The decreasing purchasing power and rising food prices during and particularly after the COVID-19 pandemic, in specific of minimally processed foods, pose a risk to increase the double burden of malnutrition documented in Mexico [36–39]. Different channels can be identified as the source of the increase in prices and inflation during the COVID-19 pandemic [58], including the disruption of the food supply chains and macroeconomic shocks [59]. Minimally processed foods, mainly fruits, vegetables and animal-source foods, are particularly sensitive to these value chain disruptions, while processed foods, UPFs and culinary ingredients may be partially buffered by storage, processing, and distribution systems [60].

Moreover, Mexico has implemented several policy measures aimed at improving dietary patterns, including taxes on sugar-sweetened beverages and non-essential energy-dense foods, front-of-package warning labels, and restrictions on marketing to children. While these policies may have increased the relative prices of some UPFs, our findings suggest that such products remain substantially cheaper per 100 kcal than minimally processed foods. This highlights the need for complementary policies that improve the affordability and price stability of minimally processed, nutrient-dense foods, including subsidies, targeted social protection programs, or investments in fresh food supply chains [61,62]. Policies that address both price levels and price volatility may be particularly important to stabilize access to nutrient-dense foods [50].

### Strengths and limitations

This study has several limitations. First, nutrient composition data were based on a food composition database and do not capture changes due to reformulation over time. Second, the use of price per 100 kcal, while informative for energy affordability, may not fully reflect consumer perceptions of food cost, which are often based on portion size or satiety [46]. In addition, our affordability metric captures price dynamics over time but does not incorporate changes in household income, wages, or purchasing power; therefore, results should be interpreted as reflecting relative food price pressures rather than true diet affordability in an income-adjusted sense. However, the study has some important strengths. It integrates nutrient density, price levels, and inflation dynamics within a single analytical framework. It uses a food-composition database developed for nationally representative dietary survey, linked with long-term price series, allowing for the examination of trends over more than a decade. The inclusion of the COVID-19 period provides an opportunity to examine how macroeconomic shocks were associated with changes in food affordability. Finally, the focus on affordable nutrient density offers a policy-relevant perspective for addressing diet quality and food security in LMICs.

### Considerations for future studies in nutrition economics

Understanding how food price inflation has affected minimally processed, processed foods, and UPFs is critical for evaluating the economic feasibility of healthy diets. Future research should further integrate food price data with nutrient profiling at both national and subnational levels. Improved linkage between retail price data from the National Geography and Statistics Institute (INEGI) and dietary and nutrient databases from the National Institute of Public Health (INSP) could enhance the monitoring of affordable nutrient density over time [2,17,63]. In addition, future studies could explore heterogeneity in price dynamics across regions, income groups, and retail environments, as well as the role of policy interventions in shaping food affordability [64]. There is also a need to examine reformulation and changes in the composition of processed foods and UPFs over time, which were not captured in the present analysis. Finally, expanding analyses to other LMIC contexts would help assess the generalizability of these findings.

## Conclusion

Minimally processed foods in Mexico were substantially more nutrient dense but were also more expensive per 100 kcal and more vulnerable to price volatility than processed foods and UPFs. Although food price inflation during and after the COVID-19 pandemic affected all categories, the higher baseline cost and greater instability of minimally processed foods widened the affordability gap between healthier and less healthy dietary options. These findings highlight a critical challenge for food systems and public health. As economic shocks increase food prices and reduce purchasing power, households may shift toward lower-cost, energy-dense foods that compromise diet quality. This dynamic has the potential to exacerbate the double burden of malnutrition, undermining efforts to reduce both obesity and micronutrient deficiencies. Integrating nutrient density with food prices over time provides a valuable framework for assessing the dynamic affordability of diets. Identifying foods that are both nutrient-rich and economically accessible can inform policies aimed at improving diet quality, food security, and health outcomes in LMICs.

## Supporting information

S1 Table Cost and inflation dynamics of nutrient-rich foods before, during, and after COVID-19 in Mexico. (DOCX)

S1 File Analytic code. (DO)

S2 File Full Spanish translation of the article. (DOCX)

S1 Data Datasets. (XLSX)
