# Supplementary material for: Rising food prices and widening nutrition gaps in Mexico: The cost of nutrient-dense and ultra-processed foods before, during, and after COVID-19
Source: PLoS One. 2026 Aug 26;21(8):e0355198. doi: 10.1371/journal.pone.0355198 (PMC13514040; doi:10.1371/journal.pone.0355198)
Supplement: Supplementary file 1 [file pone.0355198.s001.docx]

**S1 Table.** Cost and inflation dynamics of nutrient rich foods before, during, and after COVID-19 in Mexico

|  |  |  |  |  |  |  | COVID-19† | | |  |
| --- | --- | --- | --- | --- | --- | --- | --- | --- | --- | --- |
| # | NOVA††† | Food | ED  kcal/ 100g | NRF9.3 | MXN$/100 kcal (jul25) |  | Pre-Covid | During Covid | Post-  Covid |  |
| 1 | MP | Spinach | 35.20 | 140.24 | 13.81 |  | 4.61 (1.442) | 5.85 (5.721) | 11.75 (5.983) |  |
| 2 | MP | Nopal | 15.70 | 139.23 | 33.23 |  | 8.96 (11.692) | 11.84 (23.57) | 6.63  (21.634) |  |
| 3 | MP | Squash | 19.78 | 45.88 | 13.33 |  | 2.14 (14.106) | 23.22 (44.979) | 14.49  (45.855) |  |
| 4 | MP | Lettuce | 14.23 | 34.93 | 14.00 |  | 6.59 (4.318) | 9.36 (9.74) | 12.60  (11.97) |  |
| 5 | MP | Broccoli | 32.11 | 72.14 | 8.43 |  | 1.15 (1.772) | 5.85 (5.72) | 11.75  (5.983) |  |
| 6 | MP | Poblano chili | 48.00 | 239.13 | 16.03 |  | 10.38 (16.897) | 2.60 (30.87) | -2.58  (26.020) |  |
| 7 | MP | Fresh chili | 37.23 | 12.66 | 27.34 |  | 6.44 (10.395) | 0.09 (27.41) | 8.14  (14.168) |  |
| 8 | MP | Cabbage | 17.34 | 57.00 | 20.56 |  | 6.59 (4.318) | 9.36 (9.74) | 12.60  (11.967) |  |
| 9 | MP | Melon cantaloupe | 32.98 | 103.71 | 10.27 |  | 7.38 (3.124) | 12.61 (4.82) | 9.35  (3.978) |  |
| 10 | MP | Green tomato | 40.14 | 6.58 | 20.14 |  | 14.90 (24.991) | –29.01 (50.52) | 1.85  (21.759) |  |
| 11 | MP | Chicken parts | 155.00 | 1.13 | 5.74 |  | 3.84 (2.694) | 15.52 (7.07) | 6.07  (5.099) |  |
| 12 | MP | Carrot | 41.00 | 94.36 | 6.69 |  | 5.77 (4.617) | 5.04  ( 3.99) | 10.93  (14.508) |  |
| 13 | MP | Limes | 22.00 | 11.11 | 19.28 |  | 10.63 (25.183) | 51.80 (50.77) | -21.19  (31.422) |  |
| 14 | MP | Red tomato | 29.68 | 31.09 | 16.66 |  | 12.65 (23.245) | -32.86 (49.31) | 8.69  (32.360) |  |
| 15 | MP | Guava | 67.18 | 243.82 | 7.47 |  | 4.66 (6.052) | 9.70 (15.94) | 9.50  (12.987) |  |
| 16 | MP | Papaya | 43.00 | 1102.11 | 10.97 |  | 7.22 (10.544) | 7.89 (16.97) | 5.68  (18.491) |  |
| 17 | MP | Green bean | 31.06 | 23.06 | 19.77 |  | 5.48 (11.159) | 11.27 (29.06) | 6.05  (18.940) |  |
| 18 | MP | Fruit water | 23.52 | 0.58 | 6.35 |  | 7.38 (3.124) | 12.61 (4.82) | 9.35  (3.978) |  |
| 19 | MP | Grapefruit | 32.98 | 113.75 | 17.92 |  | 8.00 (7.222) | 9.27 (16.524) | 16.40  (11.957) |  |
| 20 | MP | Strawberry | 32.00 | 102.77 | 35.70 |  | 7.38 (3.124) | 12.61 (4.82) | 9.35  (3.978) |  |
| 21 | MP | Zucchini | 17.34 | 57.00 | 20.59 |  | 9.30 (14.99) | -4.67 (25.72) | 3.13  (18.006) |  |
| 22 | MP | Oranges | 47.84 | 186.60 | 12.53 |  | 5.22 (15.118) | 7.36 (29.719) | 27.67  (19.972) |  |
| 23 | MP | Jicama | 38.00 | 61.56 | 9.27 |  | 4.13 (7.163) | 8.45 (19.35) | 5.24  (12.996) |  |
| 24 | MP | Pineapple | 50.00 | 67.50 | 17.22 |  | 7.58 (7.875) | 3.72 (18.26) | 8.70  (10.240) |  |
| 25 | P | Green chili (can) | 37.03 | 12.67 | 27.34 |  | 4.81 (0.751) | 8.90 (1.466) | 10.31  (1.185) |  |
| 26 | UPF | Cereal | 377.95 | 3.78 | 3.64 |  | 4.21 (1.235) | 5.72 (2.602) | 8.52  (1.180) |  |
| 27 | UPF | Cereal light (Sp K) | 352.10 | 12.03 | 3.13 |  | 4.21 (1.235) | 5.72  (2.602) | 8.52  (1.180) |  |
| 28 | UPF | Soy drink powder | 391..23 | 57.67 | 0.59 |  | 3.95 (0.488) | 5.27  (1.342) | 4.38  (1.283) |  |
| 29 | UPF | Cereal (All bran) | 283.76 | 25.39 | 4.78 |  | 4.21 (1.235) | 5.719  (2.602) | 8.52  (1.180) |  |
| 30 | UPF | Chocolate beverage powder | 388.69 | 13.82 | 0.38 |  | 3.97 (1.133) | 7.36  (0.493) | 8.93  (0.419) |  |

† Average annualized inflation rates. Standard error in parenthesis. †† OLS estimates of β_1_ and θ defined in equation (1). ††† Minimally processed foods (MP); processed foods (P) and ultra-processed foods (UPF). * Robust standard errors (rse) in parenthesis. ‡ INPC index points.
