# Supplementary material for: Rising food prices and widening nutrition gaps in Mexico: The cost of nutrient-dense and ultra-processed foods before, during, and after COVID-19
Source: PLoS One. 2026 Aug 26;21(8):e0355198. doi: 10.1371/journal.pone.0355198 (PMC13514040; doi:10.1371/journal.pone.0355198)
Supplement: Supplementary file 2 [file pone.0355198.s003.docx]

**Aumento de los precios de los alimentos y ampliación de las brechas nutricionales en México: el costo de los alimentos densos en nutrientes y alimentos ultraprocesados antes, durante y después de COVID-19**

**Resumen**
La pandemia de COVID-19 alteró los sistemas alimentarios globales, contribuyendo al aumento de los precios de los alimentos y modificando la asequibilidad de las dietas saludables. Este estudio examinó la relación entre la densidad nutricional de los alimentos, los precios de los alimentos y la inflación en México antes, durante y después de la pandemia. Los alimentos de la encuesta ENSANUT fueron asignados a 4 categorías utilizando el sistema de clasificación NOVA y vinculados con datos de composición nutricional y series de precios de alimentos a nivel nacional de 2012 a 2025. El Nutrient Rich Food Index (NRF9.3) se utilizó como medida de densidad nutricional. La asequibilidad se definió como la densidad nutricional en relación con el precio por 100 kcal. Las tendencias en precios e inflación se analizaron utilizando un modelo de datos de panel con efectos fijos a nivel de alimento. Los alimentos mínimamente procesados presentaron una densidad nutricional sustancialmente mayor (NRF9.3: 202.2) que los alimentos procesados (15.7) y ultraprocesados (AUP; 5.4), pero también fueron considerablemente más costosos por 100 kcal. A lo largo del periodo de estudio, una mayor densidad nutricional se asoció consistentemente con precios más altos por caloría. Durante la pandemia, la inflación de los alimentos mínimamente procesados aumentó en 1.64 puntos porcentuales (p.p.; p=0.064) en relación con los niveles prepandémicos, y en 6.04 p.p. en el periodo pospandémico (p<0.001). Los alimentos procesados y los ingredientes culinarios experimentaron una inflación adicional de 1.99 p.p. y 1.21 p.p., respectivamente, durante la pandemia, mientras que no se observaron diferencias significativas entre categorías de procesamiento en el periodo pospandémico. Tras el inicio de la pandemia, los precios de los alimentos aumentaron en todas las categorías de procesamiento. Debido a que los alimentos mínimamente procesados tenían precios iniciales más elevados, tasas de inflación similares se tradujeron en una ampliación de la brecha de asequibilidad entre los alimentos más y menos nutritivos. Estos hallazgos sugieren que los alimentos alineados con las recomendaciones dietéticas se volvieron relativamente menos asequibles durante un periodo de disrupción económica, incentivando potencialmente la sustitución hacia AUP de menor costo y alta densidad energética. Las políticas que mejoren la asequibilidad y la estabilidad de precios de los alimentos densos en nutrientes podrían ser fundamentales para apoyar dietas saludables en contextos de volatilidad económica.

**Introducción**
La pandemia de COVID-19 alteró las cadenas de valor alimentarias en todo el mundo. Durante las fases iniciales de la pandemia, las medidas de contención adoptadas por los gobiernos nacionales para reducir las tasas de infección provocaron interrupciones que afectaron todas las etapas del suministro de alimentos —desde la producción y el procesamiento hasta la distribución y la venta—, lo que condujo al aumento de los precios de los alimentos y de la inflación [1]. Informes de la Organización de las Naciones Unidas para la Alimentación y la Agricultura (FAO) documentaron incrementos sustanciales en los precios globales de los alimentos tanto antes como, especialmente, durante los dos años de la pandemia [2]. Según el Fondo Monetario Internacional (FMI), el Índice Global de Precios de los Alimentos aumentó 77.26% en abril de 2022 en comparación con marzo de 2020, al inicio de la pandemia [3]. Aunque posteriormente se observó cierta corrección del mercado, los precios de los alimentos continuaron aumentando [4-6], agravados además por conflictos armados, sequías y eventos climáticos extremos. Estas tendencias económicas han afectado de manera desproporcionada a las poblaciones de países de ingresos bajos y medianos (PIBM) [7, 8].

Los precios de los alimentos en México, antes, durante y después de la pandemia, constituyen un caso de estudio relevante. Los patrones de consumo alimentario en México, al igual que en otros PIBM, están fuertemente determinados por los precios de los alimentos y por el nivel socioeconómico de los consumidores [9]. En general, la carne fresca, los lácteos, las verduras y las frutas tienen un mayor costo por 100 kcal en comparación con los cereales, las grasas y los dulces [9-12]. Los alimentos de menor costo y alta densidad energética, que proporcionan más calorías por peso, han sido más propensos a ser consumidos por poblaciones de menores ingresos y residentes en zonas rurales [9]. Además, los aumentos de precios relacionados con COVID no han sido uniformes entre los grupos de alimentos. Los precios de los alimentos frescos y perecederos (por ejemplo, carne, aves, pescado y frutas) han aumentado más pronunciadamente que los de los alimentos discrecionales [10]. Esto ha generado preocupación de que las dietas nutritivas basadas en alimentos mínimamente procesados se estén volviendo inaccesibles, no solo para las poblaciones pobres a nivel global, sino también para las poblaciones de ingresos medios [13].

El conocido sistema de clasificación NOVA [14, 15] asigna los alimentos en 4 categorías según el grado de procesamiento industrial: alimentos mínimamente procesados, alimentos procesados, alimentos ultraprocesados (AUP) e ingredientes culinarios [14]. En general, los AUP reciben puntuaciones más bajas de densidad nutricional en comparación con los alimentos mínimamente procesados [11]. La categoría de AUP, que incluye muchos alimentos envasados ricos en cereales refinados, azúcares añadidos, sodio y grasas vegetales [14, 16], se ha asociado con desenlaces adversos para la salud [14, 16].

La FAO ha enfatizado la importancia de la densidad nutricional asequible, subrayando la necesidad de identificar alimentos que sean tanto ricos en nutrientes como económicamente accesibles [17]. Los AUP suelen ser fuentes menos costosas de energía dietética, frecuentemente con un costo menor por 100 kcal que los alimentos mínimamente procesados [11, 18-21]. Sin embargo, la interacción entre el nivel de procesamiento NOVA, la densidad nutricional, los precios de los alimentos y la inflación alimentaria no ha sido suficientemente examinada. Aunque el bajo costo de los AUP ha sido documentado, con menor frecuencia se ha integrado con análisis de densidad nutricional y dinámica de precios a lo largo del tiempo.

El presente estudio tuvo como objetivo examinar la densidad nutricional y los cambios en los precios mensuales de los alimentos y en la inflación según la categoría NOVA en México antes, durante y después de la pandemia de COVID-19. Aprovechamos la variación antes, durante y después de la pandemia de COVID-19 como un entorno cuasi experimental para evaluar cambios en la inflación de los precios de los alimentos entre categorías de procesamiento NOVA y densidad nutricional. Dado que el procesamiento de alimentos puede mejorar la estabilidad de almacenamiento y la resiliencia de las cadenas de suministro [22, 23], planteamos la hipótesis de que los alimentos mínimamente procesados exhibirían una mayor variabilidad estacional y aumentos de precios más pronunciados en comparación con los alimentos procesados, los AUP y los ingredientes culinarios. Los hallazgos son relevantes para el desarrollo de guías alimentarias y para responsables de políticas públicas que abordan la inseguridad alimentaria y las enfermedades crónicas relacionadas con la dieta en contextos de PIBM.///////////////////////

**Materiales y Métodos**

**La base de datos de composición de alimentos ENSANUT 2018**

La Encuesta Nacional de Salud y Nutrición (ENSANUT) es una encuesta nacionalmente representativa sobre dietas y salud en México que incluye módulos sobre inseguridad alimentaria, ingesta dietética y desenlaces de salud [24]. Los datos de ingesta dietética se recopilan utilizando tanto un Cuestionario de Frecuencia de Consumo de Alimentos (CFCA) como un recordatorio de 24 horas. El presente estudio utilizó una base de datos de composición nutricional de los 157 productos alimenticios incluidos en el CFCA de ENSANUT. Cada alimento contenía información sobre al menos 12 nutrientes de interés. Los alimentos fueron asignados a 11 grupos de alimentos. Las hierbas y especias fueron asignadas a una categoría de “no alimentos” junto con café en polvo no reconstituido, caramelos endulzados bajos en calorías y goma de mascar. Los alimentos con una densidad energética <10 kcal/100 g fueron excluidos de los análisis de costo y densidad de nutrientes porque el NRF9.3 es una razón de nutrientes por caloría, y los alimentos con valores energéticos nulos o muy bajos (p. ej., agua, bebidas dietéticas, café y té sin azúcar) pueden dar lugar a divisiones por cero o producir estimaciones desproporcionadamente altas de densidad de nutrientes debido al pequeño denominador. La base de datos de alimentos ENSANUT se vinculó con datos sobre precios promedio de alimentos e índices de precios de alimentos proporcionados por el Instituto Nacional de Geografía y Estadística (INEGI).

**Clasificación NOVA**

El sistema NOVA clasifica los alimentos en cuatro categorías: mínimamente procesados, procesados, ultraprocesados e ingredientes culinarios, con base en el propósito e intención del procesamiento industrial [16]. Los alimentos como frutas, verduras, cereales o carnes, leche fresca y yogur natural, jugos recién exprimidos, huevos, leguminosas, pescado y otros mariscos, así como nueces y semillas sin sal listados por ENSANUT fueron clasificados como alimentos mínimamente procesados. Los ingredientes culinarios incluyeron sal, azúcar y melaza, miel, jarabe, aceites vegetales, grasas animales (manteca, mantequilla), aditivos, vinagre con aditivos y conservadores, y almidones. Los alimentos procesados incluyeron productos que combinan ingredientes culinarios con alimentos mínimamente procesados. Finalmente, los alimentos ultraprocesados (AUP) son formulaciones industriales que frecuentemente contienen altos niveles de azúcares añadidos, sodio y grasas, así como aditivos no utilizados en cocinas domésticas, como antioxidantes, saborizantes y estabilizantes [15]. Los productos clasificados en esta categoría incluyeron panes comerciales, cereales de desayuno listos para consumir, pasteles, botanas dulces, pizzas, papas fritas, helados y comidas congeladas. La Fig. 1 muestra la distribución de los alimentos ENSANUT por grupo de alimentos (A) y por categoría NOVA (B).

**Fig 1.** Distribución porcentual de los alimentos de la ENSANUT según las categorías de alimentos de WWEIA y de procesamiento de alimentos NOVA.

**Precios de alimentos y tendencias de precios**

Siguiendo procedimientos previos, los precios promedio de los 157 alimentos listados en el CFCA de ENSANUT 2018 se obtuvieron del Instituto Nacional de Geografía y Estadística (INEGI) para el mes de mayo de 2012 y se expresaron en pesos mexicanos (MXN) por 100 g o 100 ml, y por 100 kcal. Posteriormente, los precios promedio fueron ajustados por rendimiento para considerar la preparación y el desperdicio. Las series mensuales de precios de alimentos de junio de 2012 a julio de 2025 se construyeron utilizando los índices de precios de alimentos del INEGI. Todos los precios fueron ajustados por inflación utilizando los índices oficiales de precios de alimentos.

Los índices de precios mensuales estaban disponibles para 81 alimentos o grupos de alimentos. Estos índices se aplicaron a los 157 alimentos del FFQ de ENSANUT, de tal forma que el mismo índice fue asignado a los alimentos dentro de un mismo grupo alimentario. Por ejemplo, el mismo índice de precios de la leche se aplicó a distintos tipos de leche representados en ENSANUT: leche entera, leche descremada, leche semidescremada y leche de asistencia social. Otros grupos de alimentos que compartieron el mismo índice de precios fueron variedades de yogur (es decir, natural, con fruta, descremado, bebible, Danonino, etc.); quesos (es decir, fresco y Manchego); plátanos; frutas enlatadas; pollo y partes de pollo; frijoles; panes; cereales; alimentos a base de maíz; carne; y algunos grupos de frutas. Además, algunos índices de precios de alimentos solo estaban disponibles a partir de agosto de 2018 por parte del INEGI, coincidiendo con la nueva metodología del índice nacional de precios al consumidor [25]. Para completar la serie temporal de precios de estos alimentos desde mayo de 2012, utilizamos los factores mensuales de descuento de precios de los alimentos que mostraron la mayor correlación con el alimento correspondiente a partir de agosto de 2018, calculando cada precio mensual de forma retrospectiva hasta mayo de 2012. Este procedimiento permitió reconstruir series completas de precios mensuales para todos los alimentos durante todo el periodo de estudio. Para controlar los determinantes de los precios de los alimentos, también obtuvimos datos sobre precios de gas, tipos de cambio, precios internacionales de cereales y precios de combustibles a partir de la base de datos Federal Reserve Economic Data (FRED) [26].

**Evaluación de la densidad de nutrientes**

Se utilizó el modelo Nutrient Rich Food (NRF, por sus siglas en inglés) para evaluar la densidad de nutrientes de los alimentos. El puntaje NRF9.3 se calcula como la diferencia entre un subíndice de nutrientes positivos (NR9) y un subíndice de nutrientes limitantes (LIM3). El subíndice positivo NR9 se basa en el porcentaje de valores diarios (%VD) por 100 kcal para nueve nutrientes: proteína, fibra, vitamina C, potasio, vitamina A, calcio, hierro, magnesio y vitamina D. El subíndice negativo LIM se basa en el porcentaje de valores máximos recomendados (VMR) por 100 kcal para tres nutrientes a limitar: grasa saturada, azúcar añadida y sodio [27]. En ambos casos, las contribuciones de los nutrientes se limitan al 100% del valor de referencia. El NRF9.3 se calculó como:

$$NR9=\left( \frac{Nut_{inc_{1}}}{DV_{1}}+\frac{Nut_{inc_{2}}}{DV_{2}}+\ldots+\frac{Nut_{inc_{9}}}{DV_{9}} \right)$$

$$LIM3=\left( \frac{{Nut\_\lim}_{1}}{{MRV}_{1}}+\ldots+\frac{{Nut\_\lim}_{3}}{{MRV}_{3}} \right)$$

$$NRF9.3=\left[ \sum_{i=1}^{9} \frac{Nut\_inc_{i}}{\left( {DV}_{i} \right)}-\sum_{j=1}^{3} \frac{{Nut_{\lim}}_{j}}{\left( MRV_{j} \right)} \right]x(100/energ\_dens)$$

Los estándares de nutrientes se basaron en recomendaciones del Instituto Nacional de Salud Pública de México (INSP) y del Codex Alimentarius [28]. Los valores diarios de referencia (VDi) fueron: proteína (50 g), fibra (25 g), vitamina A (800 RAE), vitamina C (100 mg), vitamina D (10 mcg), calcio (1000 mg), hierro (18 mg), potasio (3500 mg) y magnesio (310 mg). Para los nutrientes a limitar, los valores máximos recomendados (VMRj) fueron: grasa saturada (20 g), azúcar añadida (50 g) y sodio (2000 mg) [28].

La densidad de nutrientes asequible, definida de manera provisional como puntos NRF9.3 por costo energético, se calculó por alimento y por grupo de alimentos NOVA. Las tendencias temporales de la densidad de nutrientes asequible se calcularon mensualmente durante el periodo de estudio mediante la siguiente fórmula:

$${Afford}_{it}=\frac{NRF9.3}{\left( \frac{MXN\$}{100 kcal} \right)}$$

El modelo NRF ha sido ampliamente utilizado en la investigación de calidad de la dieta y de los alimentos [29], y ha sido aplicado en contextos de PIBM para evaluar el valor nutricional de los alimentos en relación con su costo [30, 31]. Las herramientas de perfilamiento nutricional como el NRF son particularmente relevantes en contextos de PIBM donde coexisten el sobrepeso y la obesidad con deficiencias de micronutrientes, incluyendo ingestas inadecuadas de vitamina A, vitaminas del complejo B, folato, calcio, hierro, yodo y zinc [32].

**Análisis estadístico**

Los precios mensuales y las tasas anuales de inflación para los 157 alimentos listados en el FFQ de ENSANUT 2018 se calcularon para cada alimento para la serie temporal de mayo de 2012 a julio de 2025. Se aprovecha la variación temporal alrededor de la pandemia de COVID-19 como un choque cuasi experimental en la dinámica de precios de alimentos. Para investigar los efectos asociados con la pandemia de COVID-19 sobre la inflación de alimentos por 100 kcal, se estimó un modelo de datos de panel con efectos fijos a nivel de alimento, efectos fijos de mes y controles macroeconómicos variables en el tiempo. Se incluyeron dos indicadores de periodo (variables dicotómicas de cambio): uno para el periodo de COVID-19 (marzo de 2020 a febrero de 2022) y otro para el periodo pos-COVID-19 (marzo de 2022 a julio de 2025), utilizando el periodo pre-COVID-19 como categoría de referencia. Para capturar la estacionalidad, se incluyeron efectos fijos por mes (variables dummies estacionales). Además, se controló por factores macroeconómicos variables en el tiempo que pueden influir en la inflación de precios de alimentos, incluyendo precios del gas, tipos de cambio, precios internacionales de cereales y precios de combustibles.

Se realizó una prueba de Hausman, la cual rechazó la especificación de efectos aleatorios en favor de un modelo de efectos fijos. La especificación de efectos fijos permite controlar la heterogeneidad no observada invariante en el tiempo entre alimentos, al permitir que cada alimento tenga su propio intercepto, reduciendo así el sesgo por variables omitidas y eliminando la posible correlación entre los efectos individuales y los regresores.

El modelo de efectos fijos específico es el siguiente:

$$\ln\left( \frac{p_{it}}{p_{it-12}} \right)=\beta_{0}+\beta_{1}Covid_{t}+\beta_{2}PostC_{t}+\sum_{k=2}^{4} \delta_{k}Covid_{t}\times Nova_{k}+$$

$$+\sum_{k=2}^{4} \omega_{k}{Post}_{t}\times Nova_{k}+\beta_{3}t+\sum_{m=1}^{11} \lambda_{m}D_{m,t}+X_{t}^{'}\boldsymbol{\theta}+\alpha_{i}+\epsilon_{it}$$

donde $\beta_{0}$ representa la inflación base para los alimentos mínimamente procesados; $\beta_{1}$ y $\beta_{2}$ capturan los cambios diferenciales en la inflación para los alimentos mínimamente procesados durante y después del periodo de COVID-19, respectivamente; $\delta_{k}$ y $\omega_{k}$ capturan los cambios diferenciales en la inflación entre categorías NOVA durante y después de COVID-19, en relación con los alimentos mínimamente procesados; $\beta_{3}$ captura las tendencias lineales en el tiempo; $\lambda_{m}$ mide los efectos estacionales mensuales; θ es un vector de parámetros asociado con controles macroeconómicos variables en el tiempo (precios del gas, tipos de cambio, precios de cereales y combustibles); $\alpha_{i}$ son efectos fijos específicos por alimento; y $\epsilon_{it}$ es el término de error. El periodo base es el periodo pre-COVID-19. Los términos de interacción entre los indicadores de periodo y las categorías NOVA permiten estimar las respuestas diferenciales de la inflación según el grado de procesamiento, en relación con los alimentos mínimamente procesados. Los errores estándar se agruparon a nivel de alimento para considerar la correlación serial dentro de cada alimento a lo largo del tiempo, y la significancia estadística se evaluó mediante pruebas t, valores p y pruebas de Wald. El código analítico utilizado para los análisis está disponible en el Archivo Suplementario S1.

**Resultados**
Los alimentos incluidos en la base de datos ENSANUT (n = 157) fueron categorizados en grupos de alimentos siguiendo el esquema de codificación What We Eat in America (WWEIA) [33]. La Fig. 1 muestra la distribución por grupos de alimentos: verduras (15.9%); leches y lácteos (13.3%); proteínas (13.3%); cereales (12.6%); frutas (11.9%); platillos mixtos (10.6%); botanas y dulces (9.9%); bebidas (5.9%); grasas y aceites (3.3%); y azúcares (1.3%). De acuerdo con la clasificación NOVA, 55 alimentos (32.4%) fueron mínimamente procesados, 40 (25.8%) procesados, 57 (37.8%) ultraprocesados (AUP) y 5 (3.9%) ingredientes culinarios.

**Densidad energética y nutricional por categoría NOVA**

La Tabla 1 muestra la densidad energética (kcal/100 g), los valores del puntaje de densidad nutricional NRF9.3, los precios por 100 kcal y nuestra medida de asequibilidad para las categorías NOVA. Los alimentos mínimamente procesados presentaron la menor densidad energética (77.29 kcal/100 g), seguidos por los alimentos procesados (174.53 kcal/100 g) y los AUP (305.57 kcal/100 g). Los ingredientes culinarios tuvieron una densidad energética de 292.92 kcal/100 g. La densidad nutricional fue sustancialmente mayor en los alimentos mínimamente procesados (NRF9.3 = 202.2), en comparación con los alimentos procesados (15.7) y los AUP (5.4), mientras que los ingredientes culinarios mostraron valores intermedios (31.4).

**Tabla 1.** **Densidad energética, densidad de nutrientes (NRF9.3), precios de los alimentos (MXN$ por 100 kcal) y métricas de asequibilidad según categorías NOVA.**

|  |  | Densidad Nutricional |  | MXN$/100 kcal | | |  | Asequibilidad^b^ | | | | | |  |
| --- | --- | --- | --- | --- | --- | --- | --- | --- | --- | --- | --- | --- | --- | --- |
| Categoría de Alimentos | Densidad Energética | NRF9.3 | LIM^a^ | Pre Pandemia | Pandemia | Pos-Pandemia |  | Pre Pandemia | Pandemia | | Pos-Pandemia | |  |  |
| Mínimamente procesados | 77.29 (12.56) | 202.20 (134.42) | 0.05 (0.01) | 28.89***  (0.38) | 37.36 ***  (0.31) | 45.18***  (0.45) |  | 13.34***  (3.90) | 11.34*  (3.29) | 8.62**  (2.57) | |  | | |
| Procesados | 174.53 (15.49) | 15.65  (5.24) | 0.14 (0.02) | 3.19***  (0.04) | 4.30***  (0.04) | 5.34***  (0.05) |  | 9.38***  (3.49) | 7.79**  (2.81) | 6.23*  (2.30) | |  | | |
| Ultraprocesados | 305.57 (31.52) | 5.41  (3.52) | 0.14 (0.01) | 4.86***  (0.06) | 6.43***  (0.05) | 8.10***  (0.09) |  | 5.08**  (2.79) | 4.61**  (2.50) | 3.74  (2.08) | |  | | |
| Ingredientes culinarios | 292.92 (72.53) | 31.42  (35.22) | 0.12 (0.04) | 1.11***  (0.01) | 1.47***  (0.004) | 1.86***  (0.03) |  | 3.81  (9.56) | 3.15  (8.43) | 1.87  (6.29) | |  | | |

^a^ LIM se refiere a los tres nutrientes a limitar: grasa saturada, azúcares añadidos y sodio. ^b^ Puntos de nutrientes ajustados por caloría por peso mexicano: Asequibilidad = NRF9.3 / (MXN$ por 100 kcal). La tabla presenta valores promedio y errores estándar. *, ** y *** indican significancia estadística a los niveles del 10%, 5% y 1%, respectivamente.

**Costo por 100 kcal y tasas de inflación por categoría NOVA y alimentos densos en nutrientes**

La Fig. 2 muestra los precios mensuales promedio por categorías NOVA para toda la muestra. La Tabla 1 indica una jerarquía de precios persistente y estable a lo largo de todos los periodos de análisis, con los alimentos mínimamente procesados como los más costosos por 100 kcal, seguidos por los alimentos procesados, los AUP y los ingredientes culinarios. Este orden se mantuvo sin cambios antes, durante y después de la pandemia de COVID-19, a pesar de los aumentos generales en los precios de los alimentos en todas las categorías. A lo largo del periodo de estudio, los precios por 100 kcal aumentaron en todos los grupos NOVA tras el inicio de la pandemia. Los alimentos mínimamente procesados pasaron de MXN$28.89 en el periodo prepandémico a MXN$37.36 durante la pandemia y MXN$45.18 en el periodo pospandémico. Aunque los alimentos procesados, los AUP y los ingredientes culinarios también experimentaron incrementos sostenidos en los precios a lo largo del tiempo, se mantuvieron sustancialmente más baratos por unidad de energía durante todo el periodo. Se observó un patrón similar para la asequibilidad nutricional (NRF9.3 por MXN$). Los alimentos mínimamente procesados proporcionaron consistentemente la mayor densidad nutricional por peso monetario, aunque esta ventaja disminuyó con el tiempo (13.34 prepandemia; 11.34 durante COVID-19; 8.62 pospandemia). Los alimentos procesados y los AUP mostraron menores y decrecientes niveles de asequibilidad nutricional, lo que indica una brecha creciente en el retorno nutricional por unidad de gasto entre niveles de procesamiento.

**Fig 2. Precios promedio mensuales por categorías NOVA.** (a) Precios por 100 Kcal; (b) Precios por 100 gramos.

La Tabla S1 presenta los 30 alimentos en el cuantil más alto de densidad nutricional NRF9.3, que incluyeron alimentos mínimamente procesados, procesados y algunos AUP. Entre los alimentos mínimamente procesados se incluyeron espinaca, nopal, calabaza, brócoli, lechuga, varios tipos de chiles, tomate verde y rojo, limones, papaya, ejotes, agua de fruta, fresa, calabacita, entre otros. También se identificaron alimentos con altos puntajes NRF9.3 dentro de los alimentos procesados (por ejemplo, chile verde enlatado) y algunos AUP (por ejemplo, cereales, soya y bebidas de chocolate en polvo).

Durante la pandemia de COVID-19, los alimentos mínimamente procesados densos en nutrientes experimentaron la mayor inflación de precios. Los limones presentaron las tasas más altas de inflación mensual anualizada (51.80%), seguidos por el tomate rojo (-32.86%), tomate verde (-29.01%), calabaza (23.22%), piezas de pollo (15.52%), fresa, melón y agua de fruta (12.61%), ejote (11.27%), guayaba (9.70%), col y lechuga (9.36%), nopal (11.84%), guayaba (9.35%) y toronja (9.27%). Los alimentos con menor inflación durante la pandemia fueron chile fresco (0.09%), chile poblano (2.60%), piña (3.72%), espinaca y brócoli (5.85%), zanahoria (5.04%) y calabacita (-4.67%). Los alimentos con mayores fluctuaciones a lo largo del tiempo fueron calabaza, tomate verde, piezas de pollo, limones y tomate rojo. Las caídas más pronunciadas durante la pandemia se observaron en el tomate rojo (–32.86%) y el tomate verde (–29.01%).

Como se reporta en la Tabla 2, los alimentos procesados y los AUP mostraron, en promedio, menores tasas de inflación que los alimentos mínimamente procesados en todo el periodo, así como en los subperiodos prepandemia, durante la pandemia y pospandemia. Este patrón de ordenamiento, en el que los alimentos mínimamente procesados son más costosos que los alimentos procesados y los AUP, se mantuvo durante los tres subperiodos analizados.

**Tabla 2. Tasas de inflación e impacto de la pandemia de COVID-19 sobre la inflación de precios por categorías NOVA (por 100 kcal).**

|  |  | |  | Covid 19 | | | | |  |  |  |
| --- | --- | --- | --- | --- | --- | --- | --- | --- | --- | --- | --- |
|  | Total | |  | Pre-Pandemia | |  | Pandemia | |  | Pos-Pandemia | |
| Categoría NOVA (nT) | Media | (SE)^a^ |  | Media | (SE) ^a^ |  | Media | (SE) ^a^ |  | Media | (SE) ^a^ |
| Total alimentos | 6.98* | 0.08 |  | 5.74* | 0.10 |  | 7.73* | 0.19 |  | 9.39* | (0.15) |
| Mínimamente procesados | 8.38* | 0.19 |  | 7.37* | 0.25 |  | 8.13* | 0.49 |  | 10.86* | (0.40) |
| Procesados | 6.52* | 0.11 |  | 5.08* | 0.14 |  | 8.67* | 0.29 |  | 8.63* | (0.20) |
| Ultraprocesados | 6.11* | 0.04 |  | 4.85* | 0.04 |  | 6.68* | 0.11 |  | 8.67* | (0.10) |
| Ingredientes culinarios | 6.42* | 0.50 |  | 4.96* | 0.62 |  | 8.06* | 0.95 |  | 8.86* | (1.14) |

* Indica significancia estadística al nivel del 1%. ^a.^ Error estándard. Las estimaciones de inflación representan cambios porcentuales anualizados en los precios de los alimentos derivados de índices mensuales de precios.

**Impacto de la pandemia de COVID-19 en la inflación de alimentos**

La Tabla 3 presenta estimaciones de datos de panel con efectos fijos por alimento y por mes, controlando la heterogeneidad inobservable persistente y choques macroeconómicos comunes, incluyendo precios de la gas, tipos de cambio, precios internacionales de cereales y combustibles. El modelo evaluó las tasas de inflación anual de los alimentos en México antes, durante y después de la pandemia de COVID-19. El modelo muestra que, durante la pandemia, las tasas de inflación anual de los alimentos mínimamente procesados fueron mayores en 1.64 puntos porcentuales (p.p.) en relación con el periodo prepandémico, aunque esta estimación es solo marginalmente significativa (p = 0.064), y fueron mayores en 6.04 p.p. en el periodo pos-COVID-19 (ver estimaciones β1 y β2). Con respecto al grado de procesamiento, durante la pandemia los alimentos procesados y los ingredientes culinarios presentaron una inflación significativamente mayor en comparación con los alimentos mínimamente procesados, mientras que no se observó una diferencia estadísticamente significativa para los AUP (ver δ_i_s en la tabla). Los alimentos procesados, los AUP y los ingredientes culinarios presentaron incrementos adicionales de 1.99 p.p., 0.48 p.p. (no significativo) y 1.21 p.p., respectivamente, en relación con los alimentos mínimamente procesados. En contraste, en el periodo pospandémico los alimentos procesados, los AUP y los ingredientes culinarios no muestran diferencias significativas en inflación en comparación con los alimentos mínimamente procesados. No se observaron efectos diferenciales por grado de procesamiento NOVA en este periodo pospandémico, lo que sugiere que los cambios inflacionarios fueron similares entre categorías de procesamiento.

**Tabla 3. Impacto de la COVID-19 sobre la inflación de los alimentos (estimaciones GLS de efectos fijos, mayo de 2012 a julio de 2025).**

| Variable | Parametro | Estimación | Error Estándar Robusto* | t | P>\|t\| | [95% IC] |
| --- | --- | --- | --- | --- | --- | --- |
| *Pandemia COVID-19* | | | | | | |
| Pre | $\beta_{0}$ | 18.09 | (38.85) | 0.47 | 0.642 | (-58.64, 94.829) |
| Durante | $\beta_{1}$ | 1.64* | (0.88) | 1.86 | 0.064 | (−0.10, 3.37) |
| Pos | $\beta_{2}$ | 6.04*** | (1.69) | 3.58 | 0.000 | ( 2.70, 9.37) |
|  |  |  |  |  |  |  |
| *Interacciones durante COVID-19* | | | | | | |
| Procesado | $\delta_{2}$ | 1.99*** | (0.76) | 2.63 | 0.009 | ( 0.50, 3.49) |
| Ultraprocesado | $\delta_{3}$ | 0.49 | (0.65) | 0.75 | 0.457 | (−0.80, 1.77) |
| Ingrediente culinario | $\delta_{4}$ | 1.21** | (0.56) | 2.16 | 0.033 | ( 0.10, 2.33) |
|  |  |  |  |  |  |  |
| *Interacciones Pos-COVID-19* | | | | | | |
| Procesado | $\omega_{2}$ | 0.53 | (0.75) | 0.70 | 0.483 | (−0.95, 2.00) |
| Ultraprocesado | $\omega_{3}$ | 0.94 | (0.62) | 1.52 | 0.131 | (−0.28, 2.16) |
| Ingrediente culinario | $\omega_{4}$ | 0.78 | (0.92) | 0.84 | 0.400 | (− 1.04, 2.60) |
|  |  |  |  |  |  |  |
| Tendencia | $\beta_{3}$ | −0.05*** | (0.02) | −2.63 | 0.009 | (−0.08, −0.01) |
| *Variables de control(en ln)* |  |  |  |  |  |  |
| Precio del gas | $\theta_{1}$ | −18.68*** | (5.77) | −3.24 | 0.001 | (−25.96,−3.24) |
| Tasa de cambio | $\theta_{2}$ | 22.03*** | (4.85) | 4.54 | 0.000 | ( 12.45, 31.65) |
| Precio cereales | $\theta_{3}$ | 3.07** | (1.19) | 2.58 | 0.011 | ( 1.13, 5.75) |
| Precio del combustible | $\theta_{4}$ | 0.70 | (0.87) | 0.80 | 0.422 | (− 1.02, 2.41) |
|  |  |  |  |  |  |  |

Notas: R2(global)=0.0206; N=24,806; n=157; t=158. * Errores estándar agrupados por alimento. Pre-COVID: Mayo 2012-Febrero 2020; COVID-19: Marzo 2020-Febrero 2022; pos-COVID-19: Marzo 2022-Julio 2025. Variación entre grupos (σᵤ) = 1.31; variación dentro de los grupos (σₑ) = 11.77; rho = 0.012; prueba de Hausman p < 0.01. *, ** y *** indican significancia estadística a los niveles del 10%, 5% y 1%, respectivamente. Las estimaciones de inflación representan cambios porcentuales anualizados en los precios de los alimentos derivados de índices mensuales de precios.

Las variables de control muestran que, si bien el precio de los cereales tiene un efecto positivo y estadísticamente significativo sobre la inflación, los precios de los combustibles no muestran una asociación significativa. Los tipos de cambio tienen una asociación positiva y significativa mucho mayor, con una elasticidad estimada de 22.03%, mientras que los precios del gas muestran una asociación negativa grande y significativa (-18.68%), lo cual podría reflejar la política de control de precios implementada por el gobierno mexicano. La tendencia temporal muestra además una pequeña pero significativa disminución en la inflación (0.05 p.p. por periodo).

**Discusión**
Este estudio examinó los precios relativos y la inflación de precios de alimentos saludables (es decir, mínimamente procesados) y menos saludables (es decir, AUP) en México antes, durante y después de la pandemia de COVID-19. La pandemia de COVID-19 se utilizó como un choque cuasi experimental para examinar cambios en la inflación de precios de alimentos y la asequibilidad según la densidad nutricional y las categorías NOVA.

Se obtuvieron tres hallazgos principales. Primero, los alimentos mínimamente procesados presentaron puntajes de densidad nutricional sustancialmente más altos que los alimentos procesados y los AUP. Segundo, los alimentos con mayor puntaje de densidad nutricional también tuvieron mayores costos por 100 kcal. Tercero, los alimentos en la categoría de AUP presentaron menores puntajes de densidad nutricional, pero también menores costos por 100 kcal.

Si bien los alimentos mínimamente procesados exhibieron mayores tasas de inflación en el periodo prepandémico, la inflación durante y después de la pandemia de COVID-19 afectó de manera similar a todas las categorías NOVA. Sin embargo, dado que los alimentos mínimamente procesados tenían un mayor costo por 100 kcal en la línea de base, estos aumentos inflacionarios paralelos se tradujeron en una brecha persistente de asequibilidad. Estos hallazgos sugieren que los alimentos recomendados como saludables continúan siendo menos asequibles durante periodos de inestabilidad macroeconómica. La brecha nutricional está impulsada tanto por mayores niveles de precios iniciales de los alimentos más saludables como por presiones inflacionarias sostenidas en todas las categorías de alimentos.

Los hogares con presupuestos alimentarios limitados pueden verse obligados a comprar alimentos de menor costo y alta densidad energética con menor valor nutricional [11, 34, 35]. México ya enfrenta una doble carga de malnutrición, caracterizada por una creciente prevalencia de sobrepeso y obesidad junto con deficiencias persistentes de micronutrientes [36-39]. Las dinámicas de precios observadas pueden ayudar a explicar las desigualdades dietéticas actuales y el empeoramiento de los resultados en salud relacionados con la nutrición [40].

**Los alimentos mínimamente procesados son más densos en nutrientes pero más costosos por unidad de energía**

Este estudio confirma observaciones previas [11, 19] de que los alimentos mínimamente procesados son más densos en nutrientes que los alimentos procesados y los AUP. Las carnes, aves, pescados y productos frescos mínimamente procesados son ricos en proteínas, fibra y micronutrientes esenciales [41, 42]. En contraste, la mayoría de los AUP son energéticamente densos y contienen mayores niveles de azúcares añadidos, sodio y grasas no saludables [11, 16]. Investigaciones previas que utilizan modelos de perfil nutricional como el NRF han demostrado de manera consistente que la densidad nutricional disminuye al pasar de alimentos mínimamente procesados a procesados y ultraprocesados [11, 19].

Son relativamente pocos los estudios que han analizado simultáneamente la densidad nutricional y el costo de los AUP. En efecto, las guías alimentarias enfatizan los beneficios para la salud de los alimentos mínimamente procesados, mientras que las restricciones económicas pueden influir en la viabilidad de estas recomendaciones para los hogares. Al integrar la densidad nutricional con datos de precios e inflación, este estudio proporciona una evaluación más relevante para políticas públicas de los entornos alimentarios, destacando las barreras estructurales para el acceso a dietas saludables. Nuestros hallazgos son consistentes con análisis previos basados en datos de Estados Unidos, que muestran que los alimentos más densos en nutrientes tienen mayor costo por 100 kcal [43]. Estudios sobre el costo de las dietas también reportan que los patrones alimentarios más ricos en nutrientes, más alineados con las guías dietéticas nacionales, son más costosos, mientras que las dietas con mayor contenido de azúcares añadidos y grasas tienden a ser más baratas [44, 45].

El costo de las dietas puede medirse por 100 kcal o por unidad de densidad nutricional. El costo por 100 kcal captura los compromisos económicos que enfrentan los hogares con inseguridad alimentaria al intentar cubrir sus necesidades energéticas al menor costo posible [46]. Los alimentos que proporcionan calorías de menor costo suelen ser cereales refinados, grasas y AUP. En contraste, el costo por gramo o por porción puede llevar a conclusiones diferentes [46, 47], ya que muchos alimentos mínimamente procesados con alto contenido de agua (por ejemplo, frutas y verduras) parecen menos costosos a pesar de aportar menos calorías. Esta distinción ayuda a explicar por qué los alimentos saludables no necesariamente son más caros por unidad de peso o por porción, pero sí más costosos por 100 kcal. Desde una perspectiva de salud pública, el costo por caloría y el costo por nutriente son métricas más informativas que el costo por gramo, ya que capturan mejor la capacidad de las dietas para satisfacer tanto las necesidades energéticas como nutricionales [46].

Para complementar esta perspectiva, también examinamos la densidad nutricional por unidad de costo (utilizando nuestra medida de asequibilidad), un enfoque alineado con estudios previos [41, 48, 49]. Esta métrica ajusta la densidad nutricional por el costo energético, proporcionando una estimación de los nutrientes obtenidos por peso monetario manteniendo constante la energía. Los alimentos mínimamente procesados obtuvieron mayores puntajes en esta métrica que los alimentos procesados y los AUP, lo que indica que proporcionan más nutrientes por peso o por unidad monetaria. Sin embargo, esto no significa que los alimentos mínimamente procesados sean baratos. Bajo restricciones presupuestarias, los hogares suelen priorizar calorías de bajo costo sobre la adecuación de micronutrientes [18]. Debido a que muchos alimentos densos en nutrientes cuestan más por 100 kcal, pueden seguir siendo menos accesibles para poblaciones de bajos ingresos, a pesar de su mayor valor nutricional.

Estas diferencias económicas son críticas para las recomendaciones de salud pública. La métrica de asequibilidad captura la eficiencia nutricional condicionada a la energía, pero los hogares toman decisiones basadas en presupuestos alimentarios totales. Por lo tanto, aunque los alimentos mínimamente procesados aporten más nutrientes por unidad monetaria, su mayor costo absoluto por caloría puede limitar su consumo, especialmente en poblaciones de bajos ingresos. Esto refleja una tensión central en los sistemas alimentarios: los alimentos que son asequibles como fuente de energía no necesariamente lo son como fuente de proteína de alta calidad y micronutrientes clave. Las dietas de bajo costo pueden aliviar el hambre, pero no necesariamente cubrir los requerimientos de micronutrientes, contribuyendo al “hambre oculta” y a la mala calidad de la dieta [50].

Estudios previos han analizado la relación entre densidad nutricional y costo. Por ejemplo, los productos lácteos son una de las fuentes más económicas de calcio [42], las leguminosas proporcionan fuentes asequibles de fibra [51] y las frutas son relativamente eficientes como fuente de vitamina C [48]. Al mismo tiempo, los AUP siguen siendo una de las fuentes más baratas de energía dietética [11]. Una implicación importante es que mejorar la calidad de la dieta puede requerir cambios tanto en los precios relativos como en la asequibilidad absoluta, no solo identificar alimentos densos en nutrientes, sino también garantizar su accesibilidad financiera.

**La inflación afectó los precios de los alimentos en todas las categorías NOVA**

Los precios de los alimentos aumentaron en todas las categorías NOVA tras el inicio de la pandemia de COVID-19, en línea con datos globales [52]. Sin embargo, nuestros modelos de panel indican que las mayores diferencias en inflación entre categorías NOVA se observaron en el periodo prepandémico. Durante la pandemia y el periodo pospandémico, las presiones inflacionarias fueron ampliamente compartidas entre las cuatro categorías NOVA.

No obstante, los alimentos mínimamente procesados continuaron mostrando mayor variabilidad y volatilidad de precios, incluyendo picos más pronunciados y fluctuaciones estacionales. Esta mayor variabilidad puede reflejar su dependencia de cadenas de suministro perecederas y su exposición a choques en los sistemas de producción y distribución [53]. En contraste, los alimentos procesados y los AUP, que se benefician de mayor vida útil y cadenas de suministro más complejas, mostraron dinámicas de precios más estables en el tiempo. Dado que los alimentos mínimamente procesados partían de un precio mucho mayor por 100 kcal, tasas de inflación similares entre categorías se tradujeron en mayores costos absolutos, reforzando las brechas de asequibilidad existentes.

Es importante destacar que los aumentos mensuales de precios de alimentos estuvieron por encima de las tasas de inflación del Índice Nacional de Precios al Consumidor (INPC) del INEGI [25]. Por ejemplo, en promedio, el aumento mensual de precios para alimentos mínimamente procesados fue de 8.4% y para AUP de 6.1%, en comparación con una tasa promedio mensual de inflación del INPC de 5.0%. Estos hallazgos, junto con el deterioro del poder adquisitivo durante el periodo de la pandemia, pueden ayudar a explicar el aumento de la inseguridad alimentaria reportado en México [54].

**Economía de la nutrición y salud pública en México**

El costo de una dieta saludable se reporta como más alto en América Latina y el Caribe en comparación con cualquier otra región del mundo [2]. El índice de precios al consumidor de los países de América Latina mostró el mayor aumento (23.5%) entre diciembre de 2020 y diciembre de 2021, en comparación con 14.5% en África y 14.8% en Asia [2]. Los alimentos ricos en nutrientes de relevancia para México y otros PIBM, como hierro, vitamina A, calcio y potasio [55], tienden a ser más costosos. En los últimos años, una disminución en el consumo de alimentos mínimamente procesados, leguminosas y granos enteros [56] ha ocurrido en paralelo con un aumento en el consumo de AUP. Durante el mismo periodo, la prevalencia de obesidad ha aumentado de manera sustancial [57]. Las disrupciones económicas asociadas a la pandemia de COVID-19 pueden haber exacerbado estas tendencias.

Aunque el precio de los alimentos ricos en nutrientes no mostró un aumento diferencial durante la pandemia, estos alimentos ya eran más costosos por 100 kcal desde el inicio. La disminución del poder adquisitivo y el aumento de los precios de los alimentos durante y especialmente después de la pandemia de COVID-19, en particular de los alimentos mínimamente procesados, representan un riesgo para incrementar la doble carga de malnutrición documentada en México [36-39]. Se pueden identificar diferentes mecanismos como fuente del aumento de precios e inflación durante la pandemia de COVID-19 [58], incluyendo la disrupción de las cadenas de suministro alimentarias y choques macroeconómicos [59]. Los alimentos mínimamente procesados, principalmente frutas, verduras y alimentos de origen animal, son particularmente sensibles a estas disrupciones de las cadenas de valor, mientras que los alimentos procesados, los AUP y los ingredientes culinarios pueden estar parcialmente amortiguados por sistemas de almacenamiento, procesamiento y distribución [60].

México ha implementado diversas medidas de política pública orientadas a mejorar los patrones dietéticos, incluyendo impuestos a bebidas azucaradas y alimentos energéticamente densos no esenciales, etiquetado frontal de advertencia y restricciones a la publicidad dirigida a niños. Si bien estas políticas pueden haber aumentado los precios relativos de algunos AUP, nuestros hallazgos sugieren que estos productos siguen siendo sustancialmente más baratos por 100 kcal que los alimentos mínimamente procesados. Esto resalta la necesidad de políticas complementarias que mejoren la asequibilidad y estabilidad de precios de los alimentos densos en nutrientes, incluyendo subsidios, programas focalizados de protección social o inversiones en cadenas de suministro de alimentos frescos [61]. Las políticas que aborden tanto los niveles de precios como la volatilidad de los precios pueden ser particularmente importantes para estabilizar el acceso a alimentos densos en nutrientes [50].

**Fortalezas y limitaciones**

Este estudio tiene varias limitaciones. Primero, los datos de composición nutricional se basaron en una base de datos de alimentos y no capturan cambios debido a reformulación a lo largo del tiempo. Segundo, el uso del precio por 100 kcal, aunque informativo para la asequibilidad energética, puede no reflejar completamente las percepciones de los consumidores sobre el costo de los alimentos, que a menudo se basan en el tamaño de la porción o la saciedad [46]. Además, nuestra métrica de asequibilidad captura dinámicas de precios en el tiempo, pero no incorpora cambios en ingresos, salarios o poder adquisitivo de los hogares; por lo tanto, los resultados deben interpretarse como presiones relativas de precios de alimentos más que como asequibilidad dietética real ajustada por ingresos. Sin embargo, el estudio presenta fortalezas importantes. Integra la densidad nutricional, los niveles de precios y la dinámica inflacionaria en un solo marco analítico. Utiliza datos dietéticos representativos a nivel nacional vinculados con series de precios de largo plazo, lo que permite examinar tendencias durante más de una década. La inclusión del periodo de COVID-19 proporciona un experimento natural para evaluar el impacto de choques macroeconómicos en la asequibilidad de los alimentos. Finalmente, el enfoque en la densidad nutricional asequible ofrece una perspectiva relevante para políticas públicas en torno a la calidad de la dieta y la seguridad alimentaria en PIBM.

**Consideraciones para estudios futuros en economía de la nutrición**

Comprender cómo la inflación de precios de alimentos ha afectado a los alimentos mínimamente procesados, procesados y AUP es fundamental para evaluar la viabilidad económica de dietas saludables. Futuros estudios deberían integrar aún más los datos de precios de alimentos con el perfilamiento nutricional a nivel nacional y subnacional. Una mejor vinculación entre los datos de precios minoristas del Instituto Nacional de Geografía y Estadística (INEGI) y las bases de datos dietéticas y nutricionales del Instituto Nacional de Salud Pública (INSP) podría mejorar el monitoreo de la densidad nutricional asequible a lo largo del tiempo [2, 17, 62]. Además, futuros estudios podrían explorar la heterogeneidad en las dinámicas de precios entre regiones, grupos de ingresos y entornos minoristas, así como el papel de las intervenciones de política en la configuración de la asequibilidad de los alimentos [63]. También es necesario examinar la reformulación y los cambios en la composición de los alimentos procesados y los AUP a lo largo del tiempo, lo cual no fue capturado en el presente análisis. Finalmente, ampliar los análisis a otros contextos de países de ingresos bajos y medianos ayudaría a evaluar la generalizabilidad de estos hallazgos.

**Conclusión**
Los alimentos mínimamente procesados en México fueron sustancialmente más densos en nutrientes, pero también más costosos por 100 kcal y más vulnerables a la volatilidad de precios que los alimentos procesados y los AUP. Aunque la inflación de precios de alimentos durante y después de la pandemia de COVID-19 afectó a todas las categorías, el mayor costo inicial y la mayor inestabilidad de los alimentos mínimamente procesados ampliaron la brecha de asequibilidad entre opciones dietéticas más saludables y menos saludables. Estos hallazgos destacan un desafío crítico para los sistemas alimentarios y la salud pública. A medida que los choques económicos aumentan los precios de los alimentos y reducen el poder adquisitivo, los hogares pueden desplazarse hacia alimentos de menor costo y alta densidad energética que comprometen la calidad de la dieta. Esta dinámica tiene el potencial de exacerbar la doble carga de malnutrición, socavando los esfuerzos para reducir tanto la obesidad como las deficiencias de micronutrientes. Integrar la densidad nutricional con los precios de los alimentos a lo largo del tiempo proporciona un marco valioso para evaluar la asequibilidad dinámica de las dietas. Identificar alimentos que sean simultáneamente ricos en nutrientes y económicamente accesibles puede informar políticas orientadas a mejorar la calidad de la dieta, la seguridad alimentaria y los resultados en salud en PIBM.

**Notas de los autores**

En consonancia con los esfuerzos por promover la equidad lingüística en la investigación en salud [64], incluimos una versión completa en español de esta revisión como material suplementario para facilitar un acceso más amplio a la evidencia y fomentar su uso en políticas públicas entre audiencias no angloparlantes.
