# Supplementary material for: Rising food prices and widening nutrition gaps in Mexico: The cost of nutrient-dense and ultra-processed foods before, during, and after COVID-19
Source: PLoS One. 2026 Aug 26;21(8):e0355198. doi: 10.1371/journal.pone.0355198 (PMC13514040; doi:10.1371/journal.pone.0355198)
Supplement: Supplementary file 3 [file pone.0355198.s006.docx]

Dear PLOS ONE editor,

We thank the editor and reviewers for their careful evaluation of our manuscript. Below, we provide a point-by-point response to all comments and describe the revisions made in the manuscript. Our responses to the comments are included in blue below.

**Journal requirements:**

When submitting your revision, we need you to address these additional requirements.

1. Please ensure that your manuscript meets PLOS ONE's style requirements, including those for file naming. The PLOS ONE style templates can be found at

<https://journals.plos.org/plosone/s/file?id=wjVg/PLOSOne_formatting_sample_main_body.pdf> and 
<https://journals.plos.org/plosone/s/file?id=ba62/PLOSOne_formatting_sample_title_authors_affiliations.pdf>.

**Response:** We carefully reviewed the manuscript, supplementary materials, and file names to ensure consistency with all PLOS ONE formatting and style requirements, including the journal’s manuscript templates and file naming conventions.

2. Thank you for stating the following financial disclosure:

“AMV received funding from the Fullbright-García Robles program for visiting scholars in 2021-2022.”

Please state what role the funders took in the study.  If the funders had no role, please state: "The funders had no role in study design, data collection and analysis, decision to publish, or preparation of the manuscript." 
If this statement is not correct you must amend it as needed.

Please include this amended Role of Funder statement in your cover letter; we will change the online submission form on your behalf.

**Response:** We confirm that the funders of AMV had no role in the study design, data collection and analysis, decision to publish, or preparation of the manuscript. We have included the revised statement in the cover letter and request that this statement be added to the online submission form.

Revised Funding Statement:

“AMV received funding from the Fulbright-García Robles program for visiting scholars in 2021–2022. The funders had no role in study design, data collection and analysis, decision to publish, or preparation of the manuscript.”

3. Thank you for stating the following in the Competing Interests section:

“AD was the original developer of the Naturally Nutrient Rich (NNR) and the Nutrient Rich Food (NRF) nutrient profiling models and was or has been a member of scientific advisory panels for BEL, Lesaffre, Nestlé, FrieslandCampina, National Pork Board, and Carbohydrate Quality Panel supported by Potatoes USA. AD has worked with Ajinomoto, Ayanabio, FoodMinds, KraftHeinz, Meiji, MS-Nutrition, Nutrition Impact LLC, Nutrition Institute, PepsiCo, Samsung, and Soremantec on quantitative ways to assess nutrient density of foods.

The remaining authors declared that this work was conducted in the absence of any commercial or financial relationships that could be construed as a potential conflict of interest”

We note that one or more of the authors are employed by a commercial company:

**Clarification:** No authors are employed by a commercial company.

a.        Please provide an amended Funding Statement declaring this commercial affiliation, as well as a statement regarding the Role of Funders in your study. If the funding organization did not play a role in the study design, data collection and analysis, decision to publish, or preparation of the manuscript and only provided financial support in the form of authors' salaries and/or research materials, please review your statements relating to the author contributions, and ensure you have specifically and accurately indicated the role(s) that these authors had in your study. You can update author roles in the Author Contributions section of the online submission form.

Please also include the following statement within your amended Funding Statement. 
“The funder provided support in the form of salaries for authors [insert relevant initials], but did not have any additional role in the study design, data collection and analysis, decision to publish, or preparation of the manuscript. The specific roles of these authors are articulated in the ‘author contributions’ section.”
If your commercial affiliation did play a role in your study, please state and explain this role within your updated Funding Statement.

**Response:** No author is employed by a commercial company. AD and AMV are professors of epidemiology and economics respectively. Consulting and other activities for AD, past and present, are disclosed in the Competing Interests statement. No funding support was obtained for this study. None of the authors received any commercial funding for this research or for the manuscript. Accordingly, the Funding Statement correctly does not report any commercial funding or support for this study. AMV was supported by a Fulbright Fellowship.

b. Please also provide an updated Competing Interests Statement declaring this commercial affiliation along with any other relevant declarations relating to employment, consultancy, patents, products in development, or marketed products, etc.

**Clarification:** There is no commercial affiliation to declare. All competing interests are disclosed.

Within your Competing Interests Statement, please confirm that this commercial affiliation does not alter your adherence to all PLOS ONE policies on sharing data and materials by including the following statement: "This does not alter our adherence to  PLOS ONE policies on sharing data and materials.” (as detailed online in our guide for authors <http://journals.plos.org/plosone/s/competing-interests>) . If this adherence statement is not accurate and  there are restrictions on sharing of data and/or materials, please state these. Please note that we cannot proceed with consideration of your article until this information has been declared.

Please include both an updated Funding Statement and Competing Interests Statement in your cover letter. We will change the online submission form on your behalf.

**Response:** We have updated the Competing Interests Statement (below) to confirm that these relationships do not alter our adherence to PLOS ONE policies on sharing data and materials. The revised statement has been included in the cover letter, and we request that the online submission form be updated accordingly.

Revised Competing Interests Statement:

“AD was the original developer of the Naturally Nutrient Rich (NNR) and Nutrient Rich Food (NRF) nutrient profiling models and has served on scientific advisory panels for BEL, Lesaffre, Nestlé, FrieslandCampina, the National Pork Board, and the Carbohydrate Quality Panel supported by Potatoes USA. AD has also previously worked with Ajinomoto, Ayanabio, FoodMinds, Kraft Heinz, Meiji, MS-Nutrition, Nutrition Impact LLC, Nutrition Institute, PepsiCo, Samsung, and Soremantec on quantitative approaches to assess nutrient density of foods. These relationships are unrelated to the present work. No commercial entity provided funding, salaries, materials, or other support for this study. This does not alter our adherence to PLOS ONE policies on sharing data and materials. The remaining authors declare that they have no competing interests. There are no patents, products in development or marketed products associated with this research to declare.”

4. If the reviewer comments include a recommendation to cite specific previously published works, please review and evaluate these publications to determine whether they are relevant and should be cited. There is no requirement to cite these works unless the editor has indicated otherwise.

**Response:** The reviewers did not recommend any additional citations or previously published works for consideration.

5. Please review your reference list to ensure that it is complete and correct. If you have cited papers that have been retracted, please include the rationale for doing so in the manuscript text, or remove these references and replace them with relevant current references. Any changes to the reference list should be mentioned in the rebuttal letter that accompanies your revised manuscript. If you need to cite a retracted article, indicate the article’s retracted status in the References list and also include a citation and full reference for the retraction notice.

**Response:** We carefully reviewed the reference list to ensure that all citations are complete, accurate, and up to date. Other than the citation formatting revisions described below, no substantive changes to the reference list were necessary.

**Reviewer #1**

1. **Comment**: The manuscript presents compelling data, and the methodology as well as the structure of the writing are clear and understandable.
   The only issue I noticed is the presence of two referencing styles (including APA referencing style) within the main text. Kindly ensure that only one reference style is consistently applied throughout your manuscript.

**Response**: Thank you for this observation. We reviewed the manuscript and standardized all in-text citations and references to ensure consistency with the Vancouver referencing style required by the journal. Specifically, citations previously presented in author-date format, including Fulgoni et al. (2009), Lewis (2019), and U.S. Department of Agriculture (2018), were revised accordingly throughout the manuscript.

**Reviewer #2**

2. **Comment**: The manuscript presents a technically sound analysis using nationally representative dietary intake data linked with long-term food price series in Mexico from 2012 to 2025. The study design, conceptual framework, and analytic strategy are generally appropriate for the stated objectives. The conclusions are mostly supported by the data presented. HOWEVER, the rationale for excluding foods with energy density <10 kcal/100 g should be further justified because this may exclude nutritionally important low-calorie foods.

**Response**: Thank you for this comment. We agree that additional clarification regarding the exclusion of foods with energy density <10 kcal/100 g is needed. Foods below this threshold were excluded because the NRF9.3 is a nutrient-to-calorie ratio, and foods with zero or very low energy values can lead to division by zero or produce disproportionately high nutrient density estimates due to the small denominator. The <10 kcal/100 g cutoff was based on previous nutrient profiling research using the NRF framework.

Importantly, this exclusion primarily affected non-caloric or near non-caloric items such as water, flavored water, diet beverages, and unsweetened coffee and tea, rather than nutritionally important low-calorie foods. For example, vegetables such as lettuce and spinach (approximately 15 kcal/100 g) and skim milk (approximately 40 kcal/100 g) were retained in the analyses.

Following the reviewer’s suggestion, we revised the Methods section to clarify the rationale for this exclusion (Lines 88–91).

Revised text:

*“Foods with energy density <10 kcal/100 g were excluded from cost and nutrient density analyses because the NRF9.3 is a nutrient-to-calorie ratio, and foods with zero or very low energy values (e.g., water, diet beverages, unsweetened coffee and tea) can lead to division by zero or produce disproportionately high nutrient density estimates due to the small denominator.”*

3. **Comment**: The authors provide a detailed Data Availability Statement however, the manuscript does not clearly indicate whether the analytic code, merged dataset, or derived affordability calculations are publicly available. Sharing analytic scripts or processed datasets would improve reproducibility.

**Response**: Thank you for this important suggestion. To further enhance transparency and reproducibility, we have added the analytic code used in this study as a supplementary file (S1 File). We have also clarified the availability of the merged dataset and underlying data sources in the Data Availability Statement. Specifically, all original data sources are publicly available, and the unified analytic dataset has been deposited in a public repository and assigned a DOI.

Following the reviewer’s suggestion, we revised the Data Availability Statement as follows:

“All data used in this study are publicly available. Dietary intake data were obtained from the Mexican National Health and Nutrition Survey (ENSANUT) food frequency questionnaire (FFQ), available at: <https://ensanut.insp.mx/encuestas/ensanutcontinua2021/descargas.php>. Food price data were obtained from the National Institute of Statistics and Geography (INEGI), available at: <https://www.inegi.org.mx/app/indicesdeprecios/Estructura.aspx?idEstructura=112001300040&T=%C3%8Dndices%20de%20Precios%20al%20Consumidor&ST=INPC%20Nacional%20(mensual)>. Data on food price determinants, including gasoline prices, exchange rates, and international cereal and fuel prices, were obtained from the Federal Reserve Economic Data (FRED) database, available at: <https://fred.stlouisfed.org/tags/series?t=mexico>. The unified analytic dataset is publicly available through the Open Science Framework (OSF) repository at <https://doi.org/10.17605/OSF.IO/4QV3F>. The analytic code is included as a supplementary file.”

4. **Comment:** The manuscript is logically organized, coherent, and mostly written in clear scientific English. However, there are occasional spacing and line-break formatting artifacts likely due to manuscript conversion. These are minor and can be corrected during revision.

**Response:** Thank you for noting this issue. We carefully reviewed the manuscript and corrected spacing inconsistencies, line-break artifacts, and other minor formatting issues introduced during manuscript conversion to improve readability and presentation throughout the revised version.
