# Supplementary material for: Rising food prices and widening nutrition gaps in Mexico: The cost of nutrient-dense and ultra-processed foods before, during, and after COVID-19
Source: PLoS One. 2026 Aug 26;21(8):e0355198. doi: 10.1371/journal.pone.0355198 (PMC13514040; doi:10.1371/journal.pone.0355198)
Supplement: Peer review history

# Peer review: Decision Letter 0

**DOI:** 10.1371/journal.pone.0355198.r001

**Authors:** António Raposo

19 May 2026

PONE-D-26-19489

Rising food prices and widening nutrition gaps in Mexico: the cost of nutrient dense and ultra-processed foods before, during, and after COVID-19

PLOS One

Dear Dr. Lara-Arevalo,

Thank you for submitting your manuscript to PLOS ONE. After careful consideration, we feel that it has merit but does not fully meet PLOS ONE’s publication criteria as it currently stands. Therefore, we invite you to submit a revised version of the manuscript that addresses the points raised during the review process.

Please submit your revised manuscript by Jul 03 2026 11:59PM. If you will need more time than this to complete your revisions, please reply to this message or contact the journal office at plosone@plos.org. When you're ready to submit your revision, log on to https://www.editorialmanager.com/pone/ and select the 'Submissions Needing Revision' folder to locate your manuscript file.

Please include the following items when submitting your revised manuscript:

- A letter that responds to each point raised by the academic editor and reviewer(s). You should upload this letter as a separate file labeled 'Response to Reviewers'.
- A marked-up copy of your manuscript that highlights changes made to the original version. You should upload this as a separate file labeled 'Revised Manuscript with Track Changes'.
- An unmarked version of your revised paper without tracked changes. You should upload this as a separate file labeled 'Manuscript'.

If you would like to make changes to your financial disclosure, please include your updated statement in your cover letter. Guidelines for resubmitting your figure files are available below the reviewer comments at the end of this letter.

If applicable, we recommend that you deposit your laboratory protocols in protocols.io to enhance the reproducibility of your results. Protocols.io assigns your protocol its own identifier (DOI) so that it can be cited independently in the future. For instructions see: https://journals.plos.org/plosone/s/submission-guidelines#loc-laboratory-protocols. Additionally, PLOS ONE offers an option for publishing peer-reviewed Lab Protocol articles, which describe protocols hosted on protocols.io. Read more information on sharing protocols at https://plos.org/protocols?utm_medium=editorial-email&utm_source=authorletters&utm_campaign=protocols.

As the corresponding author, your ORCID iD is verified in the submission system and will appear in the published article. PLOS supports the use of ORCID, and we encourage all coauthors to register for an ORCID iD and use it as well. Please encourage your coauthors to verify their ORCID iD within the submission system before final acceptance, as unverified ORCID iDs will not appear in the published article. *Only*  the individual author can complete the verification step; PLOS staff *cannot*  verify ORCID iDs on behalf of authors.

We look forward to receiving your revised manuscript.

Kind regards,

António Raposo

Academic Editor

PLOS One

Journal Requirements:

When submitting your revision, we need you to address these additional requirements.

1. Please ensure that your manuscript meets PLOS ONE's style requirements, including those for file naming. The PLOS ONE style templates can be found at

https://journals.plos.org/plosone/s/file?id=wjVg/PLOSOne_formatting_sample_main_body.pdf and

https://journals.plos.org/plosone/s/file?id=ba62/PLOSOne_formatting_sample_title_authors_affiliations.pdf.

2. Thank you for stating the following financial disclosure:

“AMV received funding from the Fullbright-García Robles program for visiting scholars in 2021-2022. “

Please state what role the funders took in the study.  If the funders had no role, please state: "The funders had no role in study design, data collection and analysis, decision to publish, or preparation of the manuscript."

If this statement is not correct you must amend it as needed.

Please include this amended Role of Funder statement in your cover letter; we will change the online submission form on your behalf.

3. Thank you for stating the following in the Competing Interests section:

“AD was the original developer of the Naturally Nutrient Rich (NNR) and the Nutrient Rich Food (NRF) nutrient profiling models and was or has been a member of scientific advisory panels for BEL, Lesaffre, Nestlé, FrieslandCampina, National Pork Board, and Carbohydrate Quality Panel supported by Potatoes USA. AD has worked with Ajinomoto, Ayanabio, FoodMinds, KraftHeinz, Meiji, MS-Nutrition, Nutrition Impact LLC, Nutrition Institute, PepsiCo, Samsung, and Soremantec on quantitative ways to assess nutrient density of foods.

The remaining authors declared that this work was conducted in the absence of any commercial or financial relationships that could be construed as a potential conflict of interest”

We note that one or more of the authors are employed by a commercial company:

a.        Please provide an amended Funding Statement declaring this commercial affiliation, as well as a statement regarding the Role of Funders in your study. If the funding organization did not play a role in the study design, data collection and analysis, decision to publish, or preparation of the manuscript and only provided financial support in the form of authors' salaries and/or research materials, please review your statements relating to the author contributions, and ensure you have specifically and accurately indicated the role(s) that these authors had in your study. You can update author roles in the Author Contributions section of the online submission form.

Please also include the following statement within your amended Funding Statement.

“The funder provided support in the form of salaries for authors [insert relevant initials], but did not have any additional role in the study design, data collection and analysis, decision to publish, or preparation of the manuscript. The specific roles of these authors are articulated in the ‘author contributions’ section.”

If your commercial affiliation did play a role in your study, please state and explain this role within your updated Funding Statement.

b. Please also provide an updated Competing Interests Statement declaring this commercial affiliation along with any other relevant declarations relating to employment, consultancy, patents, products in development, or marketed products, etc.

Within your Competing Interests Statement, please confirm that this commercial affiliation does not alter your adherence to all PLOS ONE policies on sharing data and materials by including the following statement: "This does not alter our adherence to  PLOS ONE policies on sharing data and materials.” (as detailed online in our guide for authors http://journals.plos.org/plosone/s/competing-interests) . If this adherence statement is not accurate and  there are restrictions on sharing of data and/or materials, please state these. Please note that we cannot proceed with consideration of your article until this information has been declared.

Please include both an updated Funding Statement and Competing Interests Statement in your cover letter. We will change the online submission form on your behalf.

4. If the reviewer comments include a recommendation to cite specific previously published works, please review and evaluate these publications to determine whether they are relevant and should be cited. There is no requirement to cite these works unless the editor has indicated otherwise.

5. Please review your reference list to ensure that it is complete and correct. If you have cited papers that have been retracted, please include the rationale for doing so in the manuscript text, or remove these references and replace them with relevant current references. Any changes to the reference list should be mentioned in the rebuttal letter that accompanies your revised manuscript. If you need to cite a retracted article, indicate the article’s retracted status in the References list and also include a citation and full reference for the retraction notice.

[Note: HTML markup is below. Please do not edit.]

Reviewers' comments:

Reviewer's Responses to Questions

**Comments to the Author**

1. Is the manuscript technically sound, and do the data support the conclusions?

The manuscript must describe a technically sound piece of scientific research with data that supports the conclusions. Experiments must have been conducted rigorously, with appropriate controls, replication, and sample sizes. The conclusions must be drawn appropriately based on the data presented.

Reviewer #1: Yes

Reviewer #2: Partly

**********

2. Has the statistical analysis been performed appropriately and rigorously?

Reviewer #1: Yes

Reviewer #2: Yes

**********

3. Have the authors made all data underlying the findings in their manuscript fully available?

The PLOS Data policy requires authors to make all data underlying the findings described in their manuscript fully available without restriction, with rare exception (please refer to the Data Availability Statement in the manuscript PDF file). The data should be provided as part of the manuscript or its supporting information, or deposited to a public repository. For example, in addition to summary statistics, the data points behind means, medians and variance measures should be available. If there are restrictions on publicly sharing data—e.g. participant privacy or use of data from a third party—those must be specified.

Reviewer #1: Yes

Reviewer #2: No

**********

4. Is the manuscript presented in an intelligible fashion and written in standard English?

PLOS ONE does not copyedit accepted manuscripts, so the language in submitted articles must be clear, correct, and unambiguous. Any typographical or grammatical errors should be corrected at revision, so please note any specific errors here.

Reviewer #1: Yes

Reviewer #2: Yes

**********

5. Review Comments to the Author

Please use the space provided to explain your answers to the questions above. You may also include additional comments for the author, including concerns about dual publication, research ethics, or publication ethics. (Please upload your review as an attachment if it exceeds 20,000 characters)

Reviewer #1: The manuscript presents compelling data, and the methodology as well as the structure of the writing are clear and understandable.

The only issue I noticed is the presence of two referencing styles (including APA referencing style) within the main text. Kindly ensure that only one reference style is consistently applied throughout your manuscript.

Reviewer #2: The manuscript presents a technically sound analysis using nationally representative dietary intake data linked with long-term food price series in Mexico from 2012 to 2025. The study design, conceptual framework, and analytic strategy are generally appropriate for the stated objectives. The conclusions are mostly supported by the data presented. HOWEVER, the rationale for excluding foods with energy density <10 kcal/100 g should be further justified because this may exclude nutritionally important low-calorie foods.

The statistical analysis is appropriate and rigorous.

The authors provide a detailed Data Availability Statement however, the manuscript does not clearly indicate whether the analytic code, merged dataset, or derived affordability calculations are publicly available. Sharing analytic scripts or processed datasets would improve reproducibility.

The manuscript is logically organized, coherent, and mostly written in clear scientific English. However, there are occasional spacing and line-break formatting artifacts likely due to manuscript conversion.These are minor and can be corrected during revision.

**********

6. PLOS authors have the option to publish the peer review history of their article (what does this mean?). If published, this will include your full peer review and any attached files.

If you choose “no”, your identity will remain anonymous but your review may still be made public.

**Do you want your identity to be public for this peer review?**  For information about this choice, including consent withdrawal, please see our Privacy Policy.

Reviewer #1: No

Reviewer #2: **Yes:**  Chibuzor Stella Amadi

**********

[NOTE: If reviewer comments were submitted as an attachment file, they will be attached to this email and accessible via the submission site. Please log into your account, locate the manuscript record, and check for the action link "View Attachments". If this link does not appear, there are no attachment files.]

To ensure your figures meet our technical requirements, please review our figure guidelines: https://journals.plos.org/plosone/s/figures

You may also use PLOS’s free figure tool, NAAS, to help you prepare publication quality figures: https://journals.plos.org/plosone/s/figures#loc-tools-for-figure-preparation.

NAAS will assess whether your figures meet our technical requirements by comparing each figure against our figure specifications.

# Author comment: Author response to Decision Letter 1

**DOI:** 10.1371/journal.pone.0355198.r002

26 Jun 2026

We thank the editor and reviewers for their careful evaluation of our manuscript. Below, we provide a point-by-point response to all comments and describe the revisions made in the manuscript. Our responses to the comments are included in blue below.

Journal requirements:

When submitting your revision, we need you to address these additional requirements.

1. Please ensure that your manuscript meets PLOS ONE's style requirements, including those for file naming. The PLOS ONE style templates can be found at

https://journals.plos.org/plosone/s/file?id=wjVg/PLOSOne_formatting_sample_main_body.pdf and

https://journals.plos.org/plosone/s/file?id=ba62/PLOSOne_formatting_sample_title_authors_affiliations.pdf.

Response: We carefully reviewed the manuscript, supplementary materials, and file names to ensure consistency with all PLOS ONE formatting and style requirements, including the journal’s manuscript templates and file naming conventions.

2. Thank you for stating the following financial disclosure:

“AMV received funding from the Fullbright-García Robles program for visiting scholars in 2021-2022.”

Please state what role the funders took in the study. If the funders had no role, please state: "The funders had no role in study design, data collection and analysis, decision to publish, or preparation of the manuscript."

If this statement is not correct you must amend it as needed.

Please include this amended Role of Funder statement in your cover letter; we will change the online submission form on your behalf.

Response: We confirm that the funders of AMV had no role in the study design, data collection and analysis, decision to publish, or preparation of the manuscript. We have included the revised statement in the cover letter and request that this statement be added to the online submission form.

Revised Funding Statement:

“AMV received funding from the Fulbright-García Robles program for visiting scholars in 2021–2022. The funders had no role in study design, data collection and analysis, decision to publish, or preparation of the manuscript.”

3. Thank you for stating the following in the Competing Interests section:

“AD was the original developer of the Naturally Nutrient Rich (NNR) and the Nutrient Rich Food (NRF) nutrient profiling models and was or has been a member of scientific advisory panels for BEL, Lesaffre, Nestlé, FrieslandCampina, National Pork Board, and Carbohydrate Quality Panel supported by Potatoes USA. AD has worked with Ajinomoto, Ayanabio, FoodMinds, KraftHeinz, Meiji, MS-Nutrition, Nutrition Impact LLC, Nutrition Institute, PepsiCo, Samsung, and Soremantec on quantitative ways to assess nutrient density of foods.

The remaining authors declared that this work was conducted in the absence of any commercial or financial relationships that could be construed as a potential conflict of interest”

We note that one or more of the authors are employed by a commercial company:

Clarification: No authors are employed by a commercial company.

a. Please provide an amended Funding Statement declaring this commercial affiliation, as well as a statement regarding the Role of Funders in your study. If the funding organization did not play a role in the study design, data collection and analysis, decision to publish, or preparation of the manuscript and only provided financial support in the form of authors' salaries and/or research materials, please review your statements relating to the author contributions, and ensure you have specifically and accurately indicated the role(s) that these authors had in your study. You can update author roles in the Author Contributions section of the online submission form.

Please also include the following statement within your amended Funding Statement.

“The funder provided support in the form of salaries for authors [insert relevant initials], but did not have any additional role in the study design, data collection and analysis, decision to publish, or preparation of the manuscript. The specific roles of these authors are articulated in the ‘author contributions’ section.”

If your commercial affiliation did play a role in your study, please state and explain this role within your updated Funding Statement.

Response: No author is employed by a commercial company. AD and AMV are professors of epidemiology and economics respectively. Consulting and other activities for AD, past and present, are disclosed in the Competing Interests statement. No funding support was obtained for this study. None of the authors received any commercial funding for this research or for the manuscript. Accordingly, the Funding Statement correctly does not report any commercial funding or support for this study. AMV was supported by a Fulbright Fellowship.

b. Please also provide an updated Competing Interests Statement declaring this commercial affiliation along with any other relevant declarations relating to employment, consultancy, patents, products in development, or marketed products, etc.

Clarification: There is no commercial affiliation to declare. All competing interests are disclosed.

Within your Competing Interests Statement, please confirm that this commercial affiliation does not alter your adherence to all PLOS ONE policies on sharing data and materials by including the following statement: "This does not alter our adherence to PLOS ONE policies on sharing data and materials.” (as detailed online in our guide for authors http://journals.plos.org/plosone/s/competing-interests) . If this adherence statement is not accurate and there are restrictions on sharing of data and/or materials, please state these. Please note that we cannot proceed with consideration of your article until this information has been declared.

Please include both an updated Funding Statement and Competing Interests Statement in your cover letter. We will change the online submission form on your behalf.

Response: We have updated the Competing Interests Statement (below) to confirm that these relationships do not alter our adherence to PLOS ONE policies on sharing data and materials. The revised statement has been included in the cover letter, and we request that the online submission form be updated accordingly.

Revised Competing Interests Statement:

“AD was the original developer of the Naturally Nutrient Rich (NNR) and Nutrient Rich Food (NRF) nutrient profiling models and has served on scientific advisory panels for BEL, Lesaffre, Nestlé, FrieslandCampina, the National Pork Board, and the Carbohydrate Quality Panel supported by Potatoes USA. AD has also previously worked with Ajinomoto, Ayanabio, FoodMinds, Kraft Heinz, Meiji, MS-Nutrition, Nutrition Impact LLC, Nutrition Institute, PepsiCo, Samsung, and Soremantec on quantitative approaches to assess nutrient density of foods. These relationships are unrelated to the present work. No commercial entity provided funding, salaries, materials, or other support for this study. This does not alter our adherence to PLOS ONE policies on sharing data and materials. The remaining authors declare that they have no competing interests. There are no patents, products in development or marketed products associated with this research to declare.”

4. If the reviewer comments include a recommendation to cite specific previously published works, please review and evaluate these publications to determine whether they are relevant and should be cited. There is no requirement to cite these works unless the editor has indicated otherwise.

Response: The reviewers did not recommend any additional citations or previously published works for consideration.

5. Please review your reference list to ensure that it is complete and correct. If you have cited papers that have been retracted, please include the rationale for doing so in the manuscript text, or remove these references and replace them with relevant current references. Any changes to the reference list should be mentioned in the rebuttal letter that accompanies your revised manuscript. If you need to cite a retracted article, indicate the article’s retracted status in the References list and also include a citation and full reference for the retraction notice.

Response: We carefully reviewed the reference list to ensure that all citations are complete, accurate, and up to date. Other than the citation formatting revisions described below, no substantive changes to the reference list were necessary.

Reviewer #1

1. Comment: The manuscript presents compelling data, and the methodology as well as the structure of the writing are clear and understandable.

The only issue I noticed is the presence of two referencing styles (including APA referencing style) within the main text. Kindly ensure that only one reference style is consistently applied throughout your manuscript.

Response: Thank you for this observation. We reviewed the manuscript and standardized all in-text citations and references to ensure consistency with the Vancouver referencing style required by the journal. Specifically, citations previously presented in author-date format, including Fulgoni et al. (2009), Lewis (2019), and U.S. Department of Agriculture (2018), were revised accordingly throughout the manuscript.

Reviewer #2

2. Comment: The manuscript presents a technically sound analysis using nationally representative dietary intake data linked with long-term food price series in Mexico from 2012 to 2025. The study design, conceptual framework, and analytic strategy are generally appropriate for the stated objectives. The conclusions are mostly supported by the data presented. HOWEVER, the rationale for excluding foods with energy density <10 kcal/100 g should be further justified because this may exclude nutritionally important low-calorie foods.

Response: Thank you for this comment. We agree that additional clarification regarding the exclusion of foods with energy density <10 kcal/100 g is needed. Foods below this threshold were excluded because the NRF9.3 is a nutrient-to-calorie ratio, and foods with zero or very low energy values can lead to division by zero or produce disproportionately high nutrient density estimates due to the small denominator. The <10 kcal/100 g cutoff was based on previous nutrient profiling research using the NRF framework.

Importantly, this exclusion primarily affected non-caloric or near non-caloric items such as water, flavored water, diet beverages, and unsweetened coffee and tea, rather than nutritionally important low-calorie foods. For example, vegetables such as lettuce and spinach (approximately 15 kcal/100 g) and skim milk (approximately 40 kcal/100 g) were retained in the analyses.

Following the reviewer’s suggestion, we revised the Methods section to clarify the rationale for this exclusion (Lines 88–91).

Revised text:

“Foods with energy density <10 kcal/100 g were excluded from cost and nutrient density analyses because the NRF9.3 is a nutrient-to-calorie ratio, and foods with zero or very low energy values (e.g., water, diet beverages, unsweetened coffee and tea) can lead to division by zero or produce disproportionately high nutrient density estimates due to the small denominator.”

3. Comment: The authors provide a detailed Data Availability Statement however, the manuscript does not clearly indicate whether the analytic code, merged dataset, or derived affordability calculations are publicly available. Sharing analytic scripts or processed datasets would improve reproducibility.

Response: Thank you for this important suggestion. To further enhance transparency and reproducibility, we have added the analytic code used in this study as a supplementary file (S1 File). We have also clarified the availability of the merged dataset and underlying data sources in the Data Availability Statement. Specifically, all original data sources are publicly available, and the unified analytic dataset has been deposited in a public repository and assigned a DOI.

Following the reviewer’s suggestion, we revised the Data Availability Statement as follows:

“All data used in this study are publicly available. Dietary intake data were obtained from the Mexican National Health and Nutrition Survey (ENSANUT) food frequency questionnaire (FFQ), available at: https://ensanut.insp.mx/encuestas/ensanutcontinua2021/descargas.php. Food price data were obtained from the National Institute of Statistics and Geography (INEGI), available at: https://www.inegi.org.mx/app/indicesdeprecios/Estructura.aspx?idEstructura=112001300040&T=%C3%8Dndices%20de%20Precios%20al%20Consumidor&ST=INPC%20Nacional%20(mensual). Data on food price determinants, including gasoline prices, exchange rates, and international cereal and fuel prices, were obtained from the Federal Reserve Economic Data (FRED) database, available at: https://fred.stlouisfed.org/tags/series?t=mexico. The unified analytic dataset is publicly available through the Open Science Framework (OSF) repository at https://doi.org/10.17605/OSF.IO/4QV3F. The analytic code is included as a supplementary file.”

4. Comment: The manuscript is logically organized, coherent, and mostly written in clear scientific English. However, there are occasional spacing and line-break formatting artifacts likely due to manuscript conversion. These are minor and can be corrected during revision.

Response: Thank you for noting this issue. We carefully reviewed the manuscript and corrected spacing inconsistencies, line-break artifacts, and other minor formatting issues introduced during manuscript conversion to improve readability and presentation throughout the revised version.

Attachment Submitted filename: Response to Reviewers.docx

# Peer review: Decision Letter 1

**DOI:** 10.1371/journal.pone.0355198.r003

**Authors:** António Raposo

20 Jul 2026

Rising food prices and widening nutrition gaps in Mexico: the cost of nutrient dense and ultra-processed foods before, during, and after COVID-19

PONE-D-26-19489R1

Dear Dr. Lara-Arevalo,

We’re pleased to inform you that your manuscript has been judged scientifically suitable for publication and will be formally accepted for publication once it meets all outstanding technical requirements.

Within one week, you’ll receive an e-mail detailing the required amendments. When these have been addressed, you’ll receive a formal acceptance letter and your manuscript will be scheduled for publication.

An invoice will be generated when your article is formally accepted. Please note, if your institution has a publishing partnership with PLOS and your article meets the relevant criteria, all or part of your publication costs will be covered. Please make sure your user information is up-to-date by logging into Editorial Manager at Editorial Manager® and clicking the ‘Update My Information' link at the top of the page. For questions related to billing, please contact billing support.

If your institution or institutions have a press office, please notify them about your upcoming paper to help maximize its impact. If they’ll be preparing press materials, please inform our press team as soon as possible -- no later than 48 hours after receiving the formal acceptance. Your manuscript will remain under strict press embargo until 2 pm Eastern Time on the date of publication. For more information, please contact onepress@plos.org.

Kind regards,

António Raposo

Academic Editor

PLOS One

Additional Editor Comments (optional):

Reviewers' comments:

Reviewer's Responses to Questions

**Comments to the Author**

1. If the authors have adequately addressed your comments raised in a previous round of review and you feel that this manuscript is now acceptable for publication, you may indicate that here to bypass the “Comments to the Author” section, enter your conflict of interest statement in the “Confidential to Editor” section, and submit your "Accept" recommendation.

Reviewer #1: All comments have been addressed

**********

2. Is the manuscript technically sound, and do the data support the conclusions?

The manuscript must describe a technically sound piece of scientific research with data that supports the conclusions. Experiments must have been conducted rigorously, with appropriate controls, replication, and sample sizes. The conclusions must be drawn appropriately based on the data presented.

Reviewer #1: Yes

**********

3. Has the statistical analysis been performed appropriately and rigorously?

Reviewer #1: Yes

**********

4. Have the authors made all data underlying the findings in their manuscript fully available?

The PLOS Data policy requires authors to make all data underlying the findings described in their manuscript fully available without restriction, with rare exception (please refer to the Data Availability Statement in the manuscript PDF file). The data should be provided as part of the manuscript or its supporting information, or deposited to a public repository. For example, in addition to summary statistics, the data points behind means, medians and variance measures should be available. If there are restrictions on publicly sharing data—e.g. participant privacy or use of data from a third party—those must be specified.

Reviewer #1: Yes

**********

5. Is the manuscript presented in an intelligible fashion and written in standard English?

PLOS ONE does not copyedit accepted manuscripts, so the language in submitted articles must be clear, correct, and unambiguous. Any typographical or grammatical errors should be corrected at revision, so please note any specific errors here.

Reviewer #1: Yes

**********

6. Review Comments to the Author

Please use the space provided to explain your answers to the questions above. You may also include additional comments for the author, including concerns about dual publication, research ethics, or publication ethics. (Please upload your review as an attachment if it exceeds 20,000 characters)

Reviewer #1: The authors have addressed all previous concerns thoroughly and professionally. I have no further comments and consider the manuscript ready for acceptance and publication.

**********

7. PLOS authors have the option to publish the peer review history of their article (what does this mean?). If published, this will include your full peer review and any attached files.

If you choose “no”, your identity will remain anonymous but your review may still be made public.

**Do you want your identity to be public for this peer review?**  For information about this choice, including consent withdrawal, please see our Privacy Policy.

Reviewer #1: **Yes:**  Doaa Husham Majeed Alsaadi

**********

# Editor report: Acceptance letter

**DOI:** 10.1371/journal.pone.0355198.r004

**Authors:** António Raposo

PONE-D-26-19489R1

PLOS One

Dear Dr. Lara-Arevalo,

I'm pleased to inform you that your manuscript has been deemed suitable for publication in PLOS One. Congratulations! Your manuscript is now being handed over to our production team.

At this stage, our production department will prepare your paper for publication. This includes ensuring the following:

* All references, tables, and figures are properly cited

* All relevant supporting information is included in the manuscript submission,

* There are no issues that prevent the paper from being properly typeset

You will receive further instructions from the production team, including instructions on how to review your proof when it is ready. Please keep in mind that we are working through a large volume of accepted articles, so please give us a few days to review your paper and let you know the next and final steps.

Lastly, if your institution or institutions have a press office, please let them know about your upcoming paper now to help maximize its impact. If they'll be preparing press materials, please inform our press team within the next 48 hours. Your manuscript will remain under strict press embargo until 2 pm Eastern Time on the date of publication. For more information, please contact onepress@plos.org.

You will receive an invoice from PLOS for your publication fee after your manuscript has reached the completed accept phase. If you receive an email requesting payment before acceptance or for any other service, this may be a phishing scheme. Learn how to identify phishing emails and protect your accounts at https://explore.plos.org/phishing.

If we can help with anything else, please email us at customercare@plos.org.

Thank you for submitting your work to PLOS ONE and supporting open access.

Kind regards,

PLOS ONE Editorial Office Staff

on behalf of

Dr. António Raposo

Academic Editor

PLOS One
